# Astrocyte deletion of α2-Na/K ATPase triggers episodic motor paralysis in mice via a metabolic pathway

**DOI:** 10.1038/s41467-020-19915-2

**Published:** 2020-12-02

**Authors:** Sarah E. Smith, Xiaoying Chen, Lindsey M. Brier, Jonathan R. Bumstead, Nicholas R. Rensing, Alison E. Ringel, Haewon Shin, Anna Oldenborg, Jan R. Crowley, Annie R. Bice, Krikor Dikranian, Joseph E. Ippolito, Marcia C. Haigis, Thomas Papouin, Guoyan Zhao, Michael Wong, Joseph P. Culver, Azad Bonni

**Affiliations:** 1 Department of Neuroscience, Washington University School of Medicine, St. Louis, MO 63110 USA; 2 MD-PhD Program, Washington University School of Medicine, St. Louis, MO 63110 USA; 3 Department of Radiology, Washington University School of Medicine, St. Louis, MO 63110 USA; 4 Department of Biomedical Engineering, Washington University in St. Louis, St. Louis, MO 63105 USA; 5 Department of Neurology, Washington University School of Medicine, St. Louis, MO 63110 USA; 6 Department of Cell Biology, Blavatnik Institute, Harvard Medical School, Boston, MA 02115 USA; 7 Department of Internal Medicine, Washington University School of Medicine, St. Louis, MO 63110 USA; 8 Department of Physics, Washington University in St. Louis, St. Louis, MO 63105 USA

**Keywords:** Astrocyte, Migraine

## Abstract

Familial hemiplegic migraine is an episodic neurological disorder characterized by transient sensory and motor symptoms and signs. Mutations of the ion pump α2-Na/K ATPase cause familial hemiplegic migraine, but the mechanisms by which α2-Na/K ATPase mutations lead to the migraine phenotype remain incompletely understood. Here, we show that mice in which α2-Na/K ATPase is conditionally deleted in astrocytes display episodic paralysis. Functional neuroimaging reveals that conditional α2-Na/K ATPase knockout triggers spontaneous cortical spreading depression events that are associated with EEG low voltage activity events, which correlate with transient motor impairment in these mice. Transcriptomic and metabolomic analyses show that α2-Na/K ATPase loss alters metabolic gene expression with consequent serine and glycine elevation in the brain. A serine- and glycine-free diet rescues the transient motor impairment in conditional α2-Na/K ATPase knockout mice. Together, our findings define a metabolic mechanism regulated by astrocytic α2-Na/K ATPase that triggers episodic motor paralysis in mice.

## Abstract (web summary)

Mutations of α2-Na/K ATPase can cause familial hemiplegic migraine via unclear mechanisms. Here, the authors show that deletion of α2-Na/K ATPase in astrocytes results in gene expression and metabolic changes leading to cortical spreading depression and episodic transient motor paralysis in mice.

## Introduction

Familial hemiplegic migraine is an episodic neurological disorder that features transient sensory and motor aura symptoms and signs. Typically associated with headache, the transient neurological disturbances in familial hemiplegic migraine may be sufficiently severe to manifest as hemiparesis or ataxia^1–4^. Familial hemiplegic migraine episodes occur with variable though characteristically low frequency^1^. Mutations in three genes encoding ion channels, including the ion pump α2-Na/K ATPase cause familial hemiplegic migraine^5–8^, but how mutations of α2-Na/K ATPase lead to the migraine phenotype remains incompletely understood.

The ion pump Na/K ATPase is expressed in most eukaryotic cells. In addition to generating sodium and potassium ion gradients across the plasma membrane, the Na/K ATPase harbors signaling functions^9–11^. The α subunit of Na/K ATPase encodes the primary catalytic machinery of the ion pump^12–16^. Among four α isoforms, neurons predominately express α1 and α3, whereas astrocytes predominately express α2 (refs. ^17–19^). Unlike the α1 isoform, which is found uniformly within the plasma membrane, the α2 isoform in astrocytes is particularly enriched adjacent to neuronal synapses and astrocytic calcium stores^20,21^. In vitro studies suggest that depletion of α2-Na/K ATPase in astrocytes alters calcium signaling, whereas overexpression of α2-Na/K ATPase in primary astrocytes leads to altered glutamate uptake^22,23^. However, the specific in vivo roles of α2-Na/K ATPase in astrocytes in the brain have remained to be elucidated.

The pathogenesis of familial hemiplegic migraine has been investigated through analyses of patient-specific mutations in mice^24–30^. Knock-in mice with the W887R patient-specific mutation of α2-Na/K ATPase have provided insights into the effect of the mutation on α2-Na/K ATPase function^26,29^. Homozygous W887R knock-in mice fail to survive past embryogenesis, whereas heterozygous W887R knock-in mice are viable^26^. Analogously, the homozygous null mutation of α2-Na/K ATPase leads to embryonic lethality, whereas mice bearing the heterozygous null mutation of α2-Na/K ATPase are viable^31,32^. These observations buttress the conclusion from in vitro studies that the W887R mutation leads to the loss of function of α2-Na/K ATPase^8,26^. Mice bearing patient-specific mutations of α2-Na/K ATPase have also pointed to excessive glutamatergic neurotransmission in the pathogenesis of familial hemiplegic migraine^28–30^. However, knock-in mice with patient-specific α2-Na/K ATPase mutations have not displayed phenotypes of motor impairment^26,28–30^. Consistent with this observation, although abnormalities of induced cortical spreading depression (iCSD) events, considered a pathophysiological correlate of migraine aura^33,34^, have been observed in knock-in mice with patient-specific α2-Na/K ATPase mutations, these mice have not exhibited spontaneous CSD (sCSD) events^26,28^. Phenotypes of transient motor phenotypes or sCSD events relevant to familial hemiplegic migraine have been thought to be too subtle to be detected in α2-Na/K ATPase knock-in mice. On the other hand, differences in the mouse and human brain may underlie the absence of these phenotypes in knock-in mice with patient-specific mutations of α2-Na/K ATPase.

In this study, we have unexpectedly found that mice in which α2-Na/K ATPase is conditionally deleted in astrocytes display episodic transient motor paralysis. Functional neuroimaging analyses reveal that conditional knockout of α2-Na/K ATPase triggers sCSD events in the brain. The sCSD events associate with low-voltage activity (LVA) events upon EEG monitoring. Long-term video and EEG monitoring show that LVA events correlate with transient motor impairment in conditional α2-Na/K ATPase knockout mice. In mechanistic studies, transcriptomic and metabolomic analyses reveal that the loss of α2-Na/K ATPase alters metabolic gene expression in astrocytes in vivo, with consequent elevation of serine and glycine in the brain. Remarkably, feeding conditional α2-Na/K ATPase knockout mice a diet free of serine and glycine reverses the phenotype of transient motor impairment. Notably, conditional α2-Na/K ATPase knockout mice also exhibit weight loss and die prematurely during adulthood, phenotypes that are not observed in familial hemiplegic migraine, and these phenotypes are not reversed by the serine- and glycine-free diet. Collectively, our findings define a metabolic mechanism regulated by astrocytic α2-Na/K ATPase that triggers episodic transient motor paralysis in mice, with potential implications for understanding episodic neurological disorders in the human brain.

## Results

### Conditional α2-Na/K ATPase knockout mice display episodic transient paralysis

**Fig. 1:**
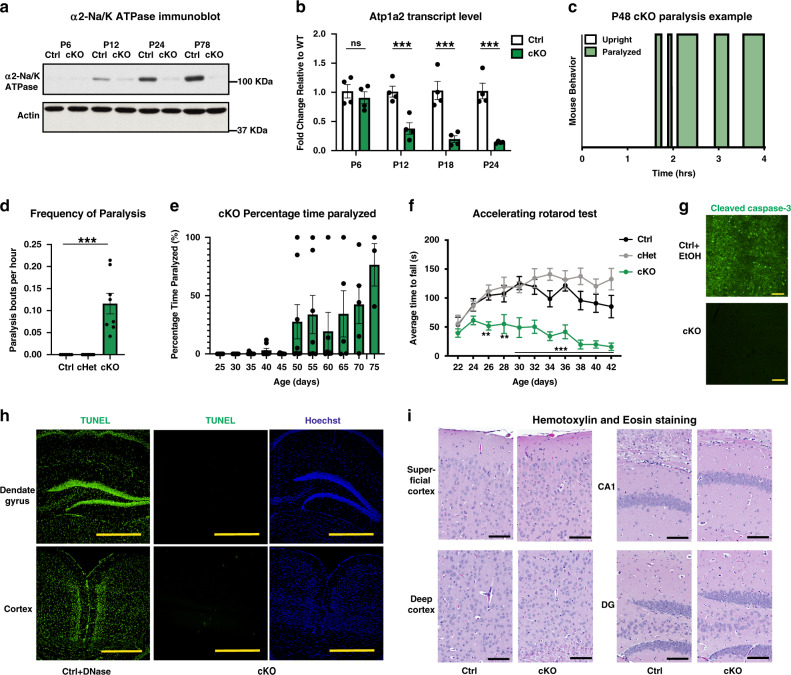
Conditional α2-Na/K ATPase knockout mice display spontaneous transient paralysis. **a** Lysates of the cerebral cortex from P6, P12, P24, and P78 conditional α2-Na/K ATPase knockout (cKO) and sex-matched littermate f/f or f/+ control (Ctrl) mice were immunoblotted with α2-Na/K ATPase and actin antibodies, the latter serving as loading control (*n* = 3 mice per group, quantification in Supplementary Fig. 2, full blots in Supplementary Fig. 15). **b** Lysates of the cerebral cortex from cKO or Ctrl mice at each age were subjected to qRT-PCR analyses using primers to α2-Na/K ATPase, normalized to Gapdh. Gapdh levels did not differ between groups (Supplementary Fig. 14a). Data are presented as mean ± s.e.m. ***P < 0.001 (two-way ANOVA followed by Fisher’s LSD post hoc test). **c**–**e** cKO, Ctrl, and conditional α2-Na/K ATPase heterozygous (cHet) mice were subjected to paralysis video monitoring. A representative trace from a P48 cKO mouse is shown (**c**). Shaded green regions represent paralysis. Quantification of video monitoring from conditional cKO, Ctrl, and cHet showing number of paralysis bouts (**d**). Note that no cHet/Ctrl mice ever exhibited an episode of paralysis. Data are presented as mean ± s.e.m. ***P < 0.001 (two-tailed *t* test, *n* = 7 Ctrl mice, 8 cHet mice, 8 cKO mice—3 male, 5 female, *P* = 0.0005). Quantification of video monitoring from cKO mice, showing total percentage time paralyzed (**e**). Data are presented as mean ± s.e.m. (*n* = 8 mice per group). **f** cKO, Ctrl, and cHet mice were subjected to an accelerating rotarod test. *P < 0.05, **P < 0.01, ***P < 0.001 (two-way ANOVA followed by Fisher’s LSD post hoc test comparing Ctrl and cKO, *n* = 6 mice per group—4 male, 2 female). **g** Representative images from cleaved caspase-3 staining from 50–75-day-old cKO and Ctrl mice. Sections from EtOH-injected Ctrl mice served as positive control. Scale bar is 100 µm; (*n* = 2). **h** Representative images of sections of the cerebral cortex from 50–75-day-old cKO and Ctrl mice subjected to TUNEL staining. DNase-treated Ctrl sections served as positive control. Scale bar is 500 µm; (*n* = 3). **i** Representative images from hematoxylin and eosin staining from 50–75-day-old cKO and Ctrl mice. Scale bar is 100 µm; (*n* = 3).

To characterize the function of α2-Na/K ATPase in the brain, we generated conditional α2-Na/K ATPase knockout mice. Because α2-Na/K ATPase is predominately expressed in astrocytes in the brain^17^, we crossed mice harboring the floxed allele of the α2-Na/K ATPase gene ATP1a2 (*α2-Na/K ATPase*^*loxP/loxP*^) with mice expressing the recombinase Cre under control of the murine GFAP (*mGFAP*) promoter. Using Cre-dependent EYFP transgenic mice, we confirmed selective expression of Cre in S100β-positive astrocytes, but not neurons in the cerebral cortex in postnatal day 20 (P20) old mice (Supplementary Fig. 1a–c). Immunoblotting and RT-qPCR analyses showed robust downregulation of α2-Na/K ATPase protein and mRNA in the cerebral cortex of conditional α2-Na/K ATPase knockout mice (Fig. 1a, b and Supplementary Fig. 2a–e). Cortical α2-Na/K ATPase protein levels were significantly reduced at P24 (17.7 ± 4.27% of Ctrl, *p* < 0.01) and modestly more reduced at P78 (4.37 ± 1.69% of Ctrl, *p* < 0.001 and *p* < 0.01 when comparing P24 and P78, Supplementary Fig. 2a, d, e). In control analyses of conditional α2-Na/K ATPase knockout mice, α2-Na/K ATPase was not downregulated in skeletal muscle (Supplementary Fig. 2f). In addition, conditional α2-Na/K ATPase knockout had little or no effect on the levels of α1-Na/K ATPase or α3-Na/K ATPase in the cerebral cortex (Supplementary Fig. 2g, h).

Conditional α2-Na/K ATPase knockout mice were born in a Mendelian ratio and appeared normal until around 1 month of age (Supplementary Fig. 3a, b). Unexpectedly, starting ~P30–P40, conditional α2-Na/K ATPase knockout mice frequently fell onto their side or abdomen and were unable to right themselves transiently for seconds to hours (Fig. 1c and Supplementary Movies 1–4). The episodes of paralysis appeared in all conditional α2-Na/K ATPase knockout mice, but not in control or conditional α2-Na/K ATPase heterozygous mice (Fig. 1d). In longitudinal video monitoring, the average amount of time of transient paralysis of conditional α2-Na/K ATPase knockout mice increased with age (Fig. 1e). Conditional α2-Na/K ATPase knockout mice also exhibited deficits in an accelerating rotarod paradigm including prior to onset of transient paralysis (Fig. 1f). Notably, conditional α2-Na/K ATPase knockout mice demonstrated significantly reduced weight beginning around the time of paralysis onset and had shortened lifespan at later ages (Supplementary Fig. 3c, d). The paralysis and lifespan phenotypes were similar in male and female conditional α2-Na/K ATPase knockout mice (Supplementary Fig. 3e–g).

Reversible paralysis caused by dysfunction of the central nervous system is typically associated with cerebrovascular ischemia or may occur in the aftermath of seizure activity^35^. No apoptotic cell death was evident in the cerebral cortex on immunohistochemical analyses, using antibodies to cleaved caspase-3 and a TUNEL assay (Fig. 1g, h). Likewise, hematoxylin and eosin (H/E) analyses revealed normal appearance of the cerebral cortex in conditional α2-Na/K ATPase knockout mice with no evidence of ischemic events (Fig. 1i). Continuous EEG monitoring, which has been successfully used to detect seizures in mice^36–38^, failed to reveal seizure activity coincident with or surrounding transient paralysis events, though one ictal event was observed in over 4000 mouse hours in conditional α2-Na/K ATPase knockout mice (Supplementary Fig. 3h). These data suggest that neither ischemia nor generalized seizures causes transient paralysis in conditional α2-Na/K ATPase knockout mice.

### Induced cortical spreading depression spreads faster in conditional α2-Na/K ATPase knockout mice

**Fig. 2:**
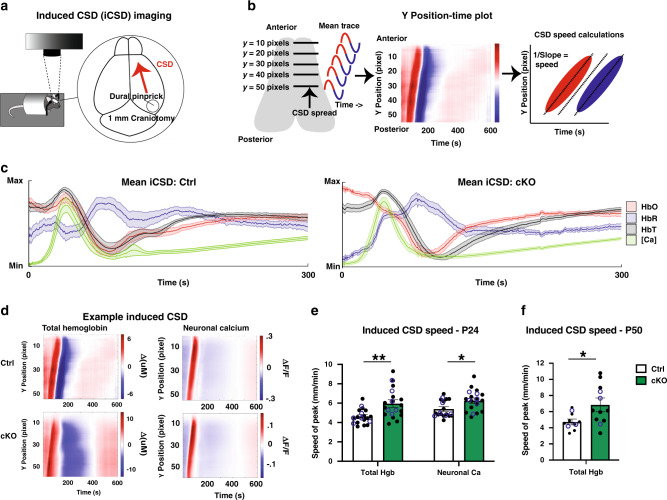
Induced cortical spreading depression spreads faster in conditional α2-Na/K ATPase knockout mice. **a**–**e** P24 conditional α2-Na/K ATPase knockout (cKO) and sex-matched littermate f/f or f/+ controls (Ctrl) mice expressing Thy1-GCaMP6f were anesthetized, subjected to a small craniotomy placed over the posterior right hemisphere and imaged for three trials after a pinprick of the dura. The average signal at each y point on the brain, for each point in time, is made into an image (position-time plot, (**d**)) where the image color represents the signal, *y*-axis represents the brain *y*-position and *x*-axis represents time (**b**). These images were used to calculate CSD speed. Traces illustrating mean ± s.e.m. for all included induced CSD trials (**c**). HbO oxygenated hemoglobin, HbR deoxygenated hemoglobin, HbT total hemoglobin, [Ca] neuronal calcium. Total time = 600 s; (*n* = 6 mice per group). Position-time plots of representative example CSD events from cKO and Ctrl animals (**d**). CSD peak spread significantly faster in the P24 cKO mice, in both the total hemoglobin and neuronal calcium traces (**e**). All iCSD position-time plots are shown in Supplementary Fig. 5 for P24. Filled in black circles represent speed for each CSD and statistics are calculated from this data, hollow blue circles represent the mean speed for each mouse. Data are presented as mean ± s.e.m. *P < 0.05, **P < 0.01 (two-tailed *t* test, *n* = 6 mice per group—3 female, 3 male and 14 CSDs in Ctrl, 15 CSDs in cKO, total Hgb = 4.61 ± 0.21 in Ctrl, 5.95 ± 0.40 in cKO, *P* = 0.030, neuronal Ca = 5.40 ± 0.24 in Ctrl, 6.25 ± 0.29 in cKO, *P* = 0.034). **f** CSD peak spread significantly faster in the hemoglobin trace for the P50 cKO mice. All iCSD position-time plots are shown in Supplementary Fig. 6 for P50. Filled in black circles represent speed for each CSD and statistics are calculated from this data, hollow blue circles represent the mean speed for each mouse. Data are presented as mean ± s.e.m. *P < 0.05 (two-tailed *t* test, *n* = 3 mice per group—2 female, 1 male and 8 CSDs in Ctrl, 9 CSDs in cKO, total Hgb = 4.74 ± 0.36 in Ctrl, 6.85 ± 0.86 in cKO, *P* = 0.047).

Because loss of-function α2-Na/K ATPase mutations cause episodic neurological symptoms and signs, including transient paralysis in patients with familial hemiplegic migraine^7^, we reasoned that episodes of paralysis in conditional α2-Na/K ATPase knockout mice might represent a phenomenon similar to migraine aura. Migraine aura is thought to be associated with cortical spreading depression (CSD), a wave of depolarization followed by hyperpolarization spreading at a slow rate (3–5 mm/min) in the cerebral cortex^39^. In animals, CSD events may be induced upon dural pinprick of the brain^40^. We developed a paradigm to image CSD in vivo using intrinsic optical signal (iOS) and neuronal calcium imaging^41^, the latter using the genetic calcium indicator GCaMP6f under control of the *Thy1* promotor (Fig. 2a). First, we showed that dural pinprick induces CSD events, and that the speed measurements for total hemoglobin and neuronal calcium closely match the speed of spread measured by EEG electrodes (Fig. 2b, Supplementary Fig. 4). Then, using the same paradigm, we observed that induced cortical spreading depression (iCSD) events were significantly increased in speed in the brain of conditional α2-Na/K ATPase knockout mice, when compared to control mice at both P24 and P50 (Fig. 2c–f, and Supplementary Figs. 5 and 6).

### Spontaneous cortical spreading depression in conditional α2-Na/K ATPase knockout mice correlates with transient motor paralysis

**Fig. 3:**
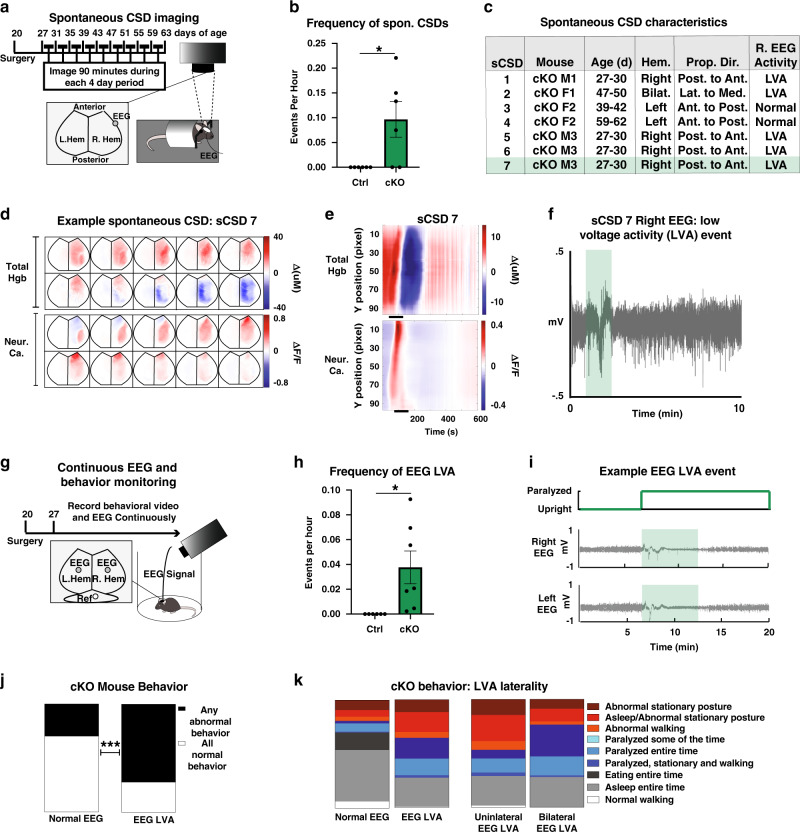
Spontaneous cortical spreading depression in conditional α2-Na/K ATPase knockout mice correlates with transient motor paralysis. **a**–**h** Conditional α2-Na/K ATPase knockout mice (cKO) and Ctrl mice were monitored for spontaneous cortical spreading depression (sCSD) events in intrinsic optical signal imaging for total hemoglobin, with GCaMP6f imaging for neuronal calcium in a subset, and right hemispheric EEG recording (*n* = 6 Ctrl, 6 cKO mice, 3 male and 3 female, 81 Ctrl and 75 cKO hours). Frequency of sCSD events in cKO and Ctrl mice (**b**). No sCSD events were observed in Ctrl mice. Data are presented as mean ± s.e.m. *P < 0.05 (two-tailed *t* test, *n* = 6 mice per group, *P* < 0.0001). Table of all observed sCSD events (**c**). LVA low-voltage activity. Green box indicates pictured sCSD7 (**d**–**f**). The position-time plots for example sCSD in **d** with propagation of signal over time is shown in **e**. The shaded box on **f** corresponds to the time captured by the still images (**d**) and the black bar (**e**, total time = 90 s, start at 50 s, 10 s between images). **g**–**k** cKO and Ctrl mice were monitored for low-voltage activity (LVA) events with continuous bilateral EEG and video monitoring. Frequency of observed LVA events in conditional cKO and Ctrl mice (**h**). No LVA events were observed in Ctrl mice. Data are presented as mean ± s.e.m. *P < 0.05 (two-tailed *t* test, *n* = 6 Ctrl mice—3 male, 3 female, 7 cKO—5 male, 2 female, *P* < 0.0001). Frequency of LVA events in cKO mice (**h**) does not differ from frequency of sCSD events (**b**, two-tailed *t* test, *P* = 0.1307). An example LVA event (**i**). Shaded box indicates LVA in right and left hemispheres, green trace indicates paralysis. LVA events in cKO mice correlated with abnormal motor behavior (**j**). Proportions of normal (eating, sleeping, and walking) and abnormal behavior (abnormal posture, abnormal walking, and paralysis) in cKO mice during normal EEG and LVA events. ***P < 0.001 (Chi-squared test, *n* = 130 events in seven mice each, *P* < 0.0001). Breakdown of behaviors during normal EEG and LVA, and unilateral vs. bilateral LVA (abnormal behavior = colors, normal behavior = grayscale, *n* = 130 events, 62 unilateral 68 bilateral, in seven mice) (**k**).

Because conditional α2-Na/K ATPase knockout mice featured the phenotype of spontaneous transient paralysis, we asked whether spontaneous cortical spreading depression (sCSD) events might arise in the brain of these mice. To capture potential sCSD events, we imaged unanesthetized conditional α2-Na/K ATPase knockout and control mice longitudinally for 90 min every 4 days using implanted cortical windows, as previously described (Fig. 3a)^41^. Strikingly, we detected robust sCSD events in conditional α2-Na/K ATPase mice, whereas no sCSD events were observed in control mice (Fig. 3b). The frequency of sCSD events in conditional α2-Na/K ATPase knockout mice was 0.10/h ± 0.04, consistent with the frequency of transient paralysis (0.12 ± 0.23, *P* = 0.644), suggesting that sCSD events might be associated with episodes of motor impairment. Notably, sCSD events included unilateral and bilateral events with distinct origins and directions of spread (Fig. 3c–f, Supplementary Fig. 7, and Supplementary Movies 5–11). Speed estimates for sCSD events yielded on average faster speeds than those measured during iCSD events (Supplementary Fig. 8). In both sCSD events and iCSD events, alterations in blood flow and neuronal activity appeared to be time-locked (Fig. 3d, e, Supplementary Fig. 6, and Supplementary Movies 5–11). Collectively, our data reveal that CSD events appear spontaneously in the brain of conditional α2-Na/K ATPase knockout mice.

Cortical spreading depression is highly associated with LVA on EEG analyses in patients^42^. EEG recordings in conditional α2-Na/K ATPase knockout mice revealed that sCSD events were also associated with periods of LVA (Fig. 3c, f and Supplementary Fig. 7). LVA EEG events were not detected outside of sCSD events in conditional α2-Na/K ATPase knockout mice or in control mice. These analyses showed that periods of LVA in EEG analyses correlated with sCSD events, suggesting that the EEG alteration might be used as a surrogate of sCSD events in conditional α2-Na/K ATPase mice.

The identification of sCSD events in awake conditional α2-Na/K ATPase knockout mice led us next to determine whether these events accompanied episodes of transient paralysis. Using implanted bilateral cortical electrodes, we recorded EEG activity and behavior simultaneously (Fig. 3g). In these analyses, periods of LVA occurred in conditional α2-Na/K ATPase knockout mice at a frequency of 0.04/h ± 0.01 (Fig. 3h), a similar frequency as sCSD events (Fig. 3b, 0.10 ± 0.04, *P* = 0.1307). Intersecting analyses of behavior with EEG telemetry, we found that periods of LVA on EEG were associated with transient abnormal motor behavior, including paralysis, abnormal stationary posture, and ataxia (Fig. 3i, j). Notably, bihemispheric LVA was strongly associated with paralysis, whereas unilateral LVA was more commonly associated with abnormal stationary posture and to a lesser extent ataxia (Fig. 3k). Together, our data suggest that sCSD events accompany transient impaired motor behavior in conditional α2-Na/K ATPase knockout mice.

### Alterations of metabolic gene expression in astrocytes in conditional α2-Na/K ATPase knockout mice

**Fig. 4:**
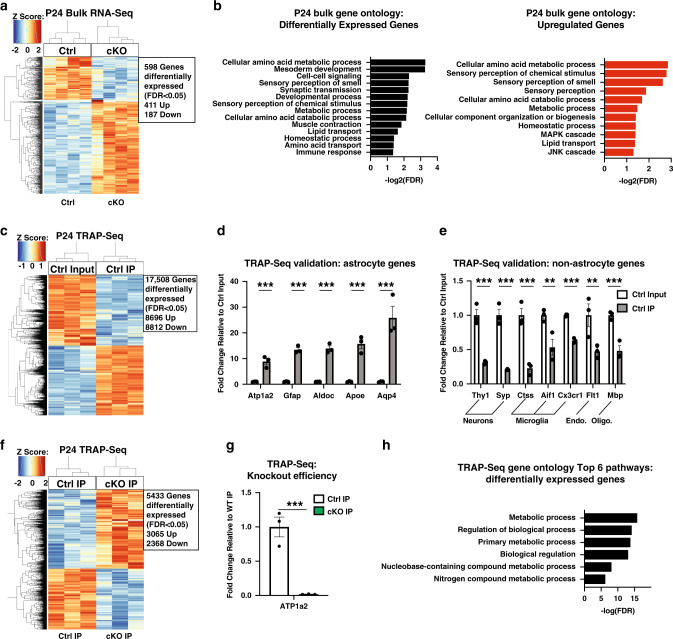
Conditional α2-Na/K ATPase knockout mice exhibit alteration of metabolic transcripts in astrocytes. **a**, **b** The cerebral cortex from P24 conditional α2-Na/K ATPase knockout (cKO) and sex-matched littermate f/f or f/+ control (Ctrl) mice were subjected to RNA-Seq analyses (*n* = 4). Clustering dendrogram and heatmap of the expression levels of significantly upregulated and downregulated genes (false discovery rate (FDR) < 0.05, *n* = 4 mice per group, *Z*-score of base 2 log-transformed CPM) (**a**). Gene ontology (GO) analyses of, from left to right, differentially expressed and upregulated genes in the P24 cKO mice (**b**). Significantly overrepresented pathways are shown (FDR < 0.05, equivalent to negative base 10 log-transformed FDR > 1.301). **c**–**h** The cerebral cortex from P24 cKO and Ctrl mice were subjected to translating ribosome affinity purification-Seq (TRAP) followed by sequencing (TRAP-Seq) analyses; (*n* = 3 mice per group). Enrichment of canonical astrocyte marker genes and de-enrichment of canonical marker genes of other cell types in the immunoprecipitation (IP) fraction of f/f or f/+ Ctrl mice, using an antibody to GFP, relative to input for TRAP-Seq (**d**, **e**). Data are presented as mean ± s.e.m. ***P < 0.001 (two-tailed *t* test, *n* = 3 mice per group). Clustering dendrogram and heatmap of the expression levels of significantly upregulated and downregulated genes in the IP fraction from cKO mice compared to the IP fraction from sex-matched Ctrl mice (**f**) (FDR < 0.05, *n* = 3 mice per group, *Z*-score of base 2 log-transformed CPM). Atp1a2 transcript levels in Ctrl and cKO IP samples (**g**). Data are presented as mean ± s.e.m. ***P < 0.001 (*t* test, *n* = 3 mice per group, *P* = 0.0007). Top six pathways (by FDR) for GO analyses of differentially expressed genes in the P24 cKO mice relative to sex-matched littermate Ctrl mice (**h**). Significantly overrepresented pathways are shown (FDR < 0.05, equivalent to negative base 10 log-transformed FDR > 1.301).

Having characterized an association between CSD and transient paralysis in conditional α2-Na/K ATPase knockout mice, we next determined a mechanism by which loss of α2-Na/K ATPase leads to the pathogenesis of motor impairment in these mice. We first performed RNA-Seq analyses in the cerebral cortex in conditional α2-Na/K ATPase knockout and control mice at P24, prior to onset of the phenotype of transient paralysis (Fig. 1e). We found nearly 600 deregulated (411 upregulated and 187 downregulated) genes in the cerebral cortex upon conditional knockout of α2-Na/K ATPase (Fig. 4a and Supplementary Data 1). Gene ontology (GO) analyses revealed that metabolism genes were overrepresented among the upregulated genes in P24 conditional α2-Na/K ATPase knockout mice (Fig. 4b). The astrocytosis gene Gfap was also upregulated in the brain of conditional α2-Na/K ATPase knockout mice (Supplementary Fig. 9a). Gfap protein upregulation was confirmed in immunohistochemical analyses, and other astrocytosis genes were also upregulated on RT-qPCR (Supplementary Fig. 9b, c)^43^. Remarkably, RNA-Seq and RT-qPCR analyses of the cerebral cortex in P17 mice revealed deregulation of metabolism, but not astrocytosis genes (Supplementary Fig. 9d–f and Supplementary Data 1), suggesting loss of α2-Na/K ATPase leads to alterations of metabolic gene expression prior to onset of astrocytosis in conditional α2-Na/K ATPase knockout mice. Among the most upregulated metabolism genes at P17 were those encoding a glycine synthesis enzyme, a mitochondrial citric acid cycle enzyme, and glutamate transaminase (Supplementary Fig. 9f).

To determine whether the deregulation of metabolic gene expression occurs in a cell-intrinsic manner in conditional α2-Na/K ATPase knockout mice, we performed translating ribosome affinity purification (TRAP) analyses of the cerebral cortex from mice, in which the EGFP/Rpl10a fusion protein was expressed in a Cre-dependent manner using the mGFAP-Cre driver. TRAP analyses in control mice led to the enrichment of genes selectively expressed in astrocytes and de-enrichment for genes expressed in neurons, microglia, and endothelial cells (Fig. 4c–e), validating the TRAP approach. Expression of α2-Na/K ATPase mRNA was nearly completely (98.2%) downregulated in TRAP analyses of conditional α2-Na/K ATPase knockout mice (Fig. 4f, g), confirming knockout of α2-Na/K ATPase in astrocytes. Importantly, metabolic genes including those that encode amino acid metabolism were deregulated in astrocytes in the cerebral cortex in conditional α2-Na/K ATPase knockout mice (Fig. 4h and Supplementary Data 1). Together, our data show that metabolic gene expression is deregulated in astrocytes in conditional α2-Na/K ATPase knockout mice.

### Levels of serine and glycine are elevated in the cerebral cortex in conditional α2-Na/K ATPase knockout mice

**Fig. 5:**
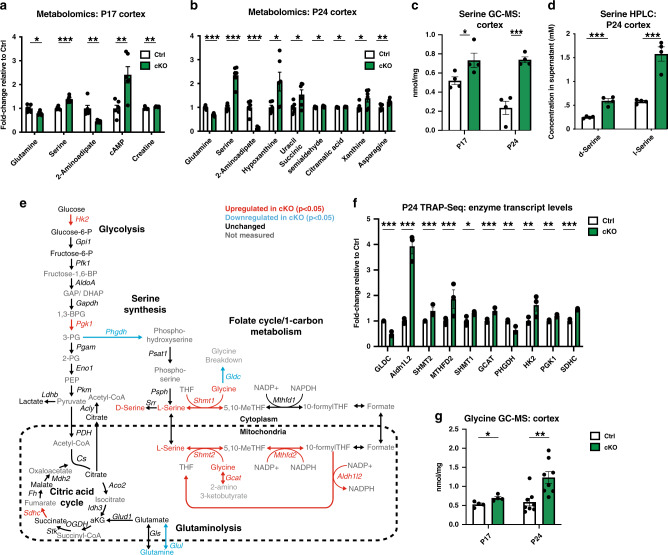
Levels of serine and glycine are elevated in the cerebral cortex of conditional α2-Na/K ATPase knockout mice. **a** Metabolomics was performed on flash-frozen cerebral cortex from P17 conditional α2-Na/K ATPase knockout (cKO) and sex-matched littermate f/f or f/+ control (Ctrl) mice. Only significantly altered metabolites are shown. Data are presented as mean ± s.e.m. *P < 0.05, **P < 0.01, ***P < 0.001 (two-tailed *t* test, *n* = 6 mice per group). **b** Metabolomics was performed on flash-frozen cerebral cortex from P24 cKO and Ctrl mice. Only significantly altered metabolites are shown. Data are presented as mean ± s.e.m. *P < 0.05, **P < 0.01, ***P < 0.001 (two-tailed *t* test, *n* = 6 mice per group). **c** The cerebral cortex from perfused P24 and P17 cKO and Ctrl mice was subjected to gas chromatography–mass spectrometry (GC–MS) for serine levels. The levels of serine were robustly elevated in the cerebral cortex of conditional α2-Na/K ATPase knockout mice. Data are presented as mean ± s.e.m. *P < 0.05, ***P < 0.001 (two-tailed *t* test, *n* = 4 mice per group, *P* = 0.046 P17, *P* = 0.00056 P24). **d** Flash-frozen cerebral cortex from P24 cKO and Ctrl mice was subjected to high-performance liquid chromatography (HPLC) to quantify levels of D- and L-serine. Data are presented as mean ± s.e.m. ***P < 0.001 (two-tailed *t* test, *n* = 4 mice per group, *P* = 0.00034 D-serine, *P* = 0.00058 L-serine). **e** Network constructed by integrating established metabolic pathways relevant to serine biosynthesis with TRAP-Seq data for transcript levels of metabolic enzymes and the metabolomics data for metabolite levels. Red indicates significant upregulation (*P* < 0.05), blue indicates significant downregulation, black indicates no significant change, and gray indicates an unmeasured metabolite. **f** Fold change of selected metabolic enzymes in the TRAP-Seq for cKO relative to Ctrl mice. Data are presented as mean ± s.e.m. *P < 0.05, **P < 0.01, ***P < 0.001 (false discovery rate (FDR), *n* = 3 mice per group). **g** The cerebral cortex from perfused P24 and P17 cKO and Ctrl mice was subjected to GC–MS for glycine levels. The levels of glycine were robustly elevated in the cerebral cortex of conditional α2-Na/K ATPase knockout mice. Data are presented as mean ± s.e.m. *P < 0.05, **P < 0.01 (two-tailed *t* test, *n* = 4 mice at P17, 8 at P24, *P* = 0.020 P17, *P* = 0.0035 P24).

To elucidate the consequences of deregulated metabolic gene expression in the brain upon loss of α2-Na/K ATPase, we performed metabolomics analyses in the cerebral cortex of conditional α2-Na/K ATPase knockout and control mice. Strikingly, the amino acid serine was the most significantly altered metabolite in the cerebral cortex upon conditional knockout of α2-Na/K ATPase in mice at both P17 and P24, increasing more than twofold at P24 (Fig. 5a, b). Other amino acids, including notably glutamate, were not significantly altered, though glutamine was slightly downregulated (Supplementary Fig. 10a–c). Targeted gas chromatography–mass spectrometry (GC–MS) confirmed upregulation of serine in the cerebral cortex, but not liver, in conditional α2-Na/K ATPase mice (Fig. 5c and Supplementary Fig. 10d). Unlike in conditional knockout mice, conditional heterozygous mice exhibited no significant increase in cortical serine levels (Supplementary Fig. 10e). In high-performance liquid chromatography (HPLC) assays, both L- and D-enantiomers of serine were equivalently upregulated in the brain of conditional α2-Na/K ATPase mice (Fig. 5d).

To shed light on the mechanisms by which loss of α2-Na/K ATPase triggers the elevation of serine levels in the brain, we constructed a network by integrating established metabolic pathways relevant to serine biosynthesis with our TRAP-Seq data (Fig. 5e). These analyses revealed that several significantly upregulated enzymes represent components of the folate cycle, which relies on the interconversion of serine and glycine for carbon units^44^. These genes included serine hydroxymethyltransferase 1 and 2, aldehyde dehydrogenase 1 family member L2, glycine C-acetyltransferase, and methylenetetrahydrofolate dehydrogenase 2 (Shmt2, Shmt1, Aldh1l2, Gcat, and Mthfd1, Fig. 5e, f). Enzymes that promote serine biosynthesis from glucose, such as phosphoglycerate dehydrogenase, phosphoserine aminotransferase 1, and phosphoserine phosphatase were downregulated or unaltered (Fig. 5e, f). Because glycine is intimately involved with serine in the pathways altered upon loss of α2-Na/K ATPase (Fig. 5e), we performed GC–MS analyses of glycine in conditional α2-Na/K ATPase knockout mice. Like serine, glycine was upregulated in the cerebral cortex, but not liver, of conditional α2-Na/K ATPase knockout mice (Fig. 5g and Supplementary Fig. 10f).

Because Na/K ATPase activity may influence mitochondrial metabolism via altered calcium dynamics^11,22,45^, and mitochondrial dysfunction deregulates serine biosynthesis^46^, we asked whether loss of α2-Na/K ATPase in the conditional knockout mice might impact mitochondrial function. We first characterized the morphology of mitochondria in astrocytes in the cerebral cortex in P24 conditional α2-Na/K ATPase knockout mice (Supplementary Fig. 11a). A modest but significant increase in the proportion of mitochondria with abnormal morphology was evident in the cortex upon conditional knockout of α2-Na/K ATPase (Supplementary Fig. 11a). In other analyses, mitochondrial genes were significantly enriched among the deregulated, mostly upregulated genes in astrocytes obtained in TRAP-Seq analyses upon intersection with the MitoCarta database of 1158 verified mitochondrial genes (Supplementary Fig. 11b, c and Supplementary Data 1)^47^. The upregulated mitochondrial function transcripts in conditional α2-Na/K ATPase knockout mice encoded serine and glycine biosynthesis, including Shmt2, Mthfd2 and Aldh1l2, and Gatm (Supplementary Fig. 11d and Supplementary Data 1). Construction of a network encompassing all metabolites significantly altered at P24 or P17 (Fig. 5a, b) with TRAP-Seq gene expression changes showed that altered metabolites participate in mitochondrial pathways, including the citric acid cycle and folate/1-carbon metabolism, or in purine and pyrimidine salvage pathways, which occur in the cytoplasm but are dependent on mitochondrial folate cycle cofactors (Supplementary Fig. 11e). Taken together, our results suggest that loss of α2-Na/K ATPase leads to alterations of mitochondrial metabolism, and elevation of levels of serine and glycine in the brain.

### Serine- and glycine-free diet reverses episodic transient motor paralysis in conditional α2-Na/K ATPase knockout mice

**Fig. 6:**
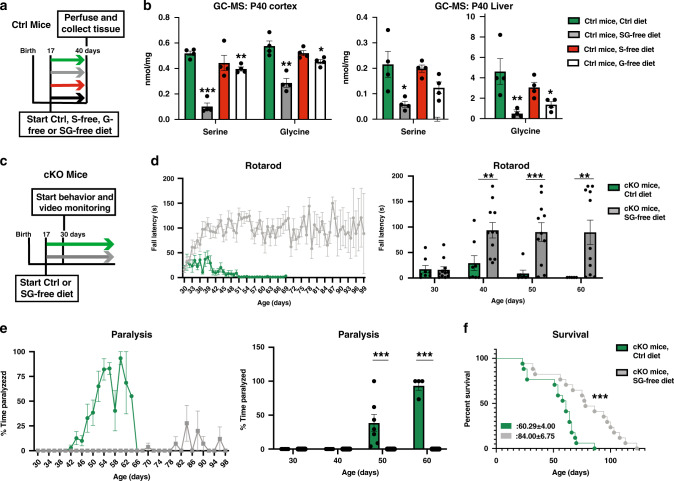
Serine- and glycine-free diet reverses motor paralysis and ataxia in conditional α2-Na/K ATPase knockout mice. **a**, **b** Sex-matched littermate f/f or f/+ control (Ctrl) mice were weaned at P17 onto one of the following diets: Ctrl, serine-free (S-free), glycine-free (G-free), or serine- and glycine-free (SG-free). The cerebral cortex and liver collected from P40 perfused Ctrl mice on Ctrl, S-free, G-free, and SG-free food were subjected to GC–MS for serine and glycine levels (**b**). The SG-free diet reduced the levels of serine and glycine substantially in the cerebral cortex and liver in Ctrl mice. Data are presented as mean ± s.e.m. *P < 0.05, **P < 0.01, ***P < 0.001 (two-tailed *t* test, *n* = 4 mice per group). **c**–**e** Sex-matched littermate conditional α2-Na/K ATPase knockout (cKO) mice were weaned onto Ctrl or SG-free diet at P17 and weighed daily thereafter. Daily rotarod analyses and every other day monitoring for motor paralysis of mice was begun at P30. Left: mean latency to fall on constant velocity rotarod task for cKO mice on Ctrl or SG-free diet (**d**). Data are presented as mean ± s.e.m. (*n* = 11 mice per group starting at P17). Right: a subset of data from left side shown for clarity. Data are presented as mean ± s.e.m. **P < 0.01, ***P < 0.001 (two-way ANOVA followed by Fisher’s LSD post hoc test, *n* = 11 mice per group starting at P17—4 males, 7 females). Left: percentage of time paralyzed for sex-matched littermate conditional α2-Na/K ATPase knockout mice (cKO) on Ctrl or SG-free food (**e**). Data are presented as mean ± s.e.m. ***P < 0.001 (two-tailed *t* test, *n* = 11 mice per group starting at P17). Right: a subset of data from left side shown for clarity. The serine- and glycine-free diet reversed transient motor paralysis in conditional α2-Na/K ATPase knockout mice. Data are presented as mean ± s.e.m. ***P < 0.001 (two-way ANOVA followed by Fisher’s LSD post hoc test, *n* = 11 mice per group starting at P17—4 males, 7 females). **f** Survival was modestly, albeit significantly, extended in cKO mice on SG-free food compared to Ctrl food. ***P < 0.001 (log-rank (Mantel–Cox) test, *n* = 17 mice per group—6 males, 11 females, *P* = 0.0008).

To determine the significance of elevation of serine and glycine levels in the pathogenesis of transient motor paralysis, we assessed the effect of lowering the levels of these amino acids on the motor phenotype in conditional α2-Na/K ATPase mice. Beginning at P17, we fed control mice one of four diets: control diet, serine-free diet, glycine-free diet, or serine- and glycine-free diet (Fig. 6a). Unlike the serine-free and glycine-free diets, the diet free of both serine and glycine substantially reduced the levels of serine and glycine in the cerebral cortex and liver in control mice (Fig. 6b). Conditional α2-Na/K ATPase mice fed the serine- and glycine-free diet likewise exhibited reduced levels of cortical and liver serine and glycine (Supplementary Fig. 12a, b). Conditional α2-Na/K ATPase knockout mice fed the control or serine- and glycine-free diet were both viable with no significant differences in their weight (Supplementary Fig. 12c, d). However, in analyses of motor behavior using the rotarod paradigm, the serine- and glycine-free diet dramatically improved the motor performance of conditional α2-Na/K ATPase mice (Fig. 6c, d). Strikingly, whereas conditional α2-Na/K ATPase knockout mice on control diet displayed episodic paralysis, these mice displayed no bouts of transient paralysis when fed the serine- and glycine-free diet (Fig. 6e). The remarkable salutary effect of the serine- and glycine-free diet in conditional α2-Na/K ATPase mice was evident >1 month after onset of the motor phenotype in the control diet cohort (Fig. 6e). When followed until their death, conditional α2-Na/K ATPase knockout mice on the serine- and glycine-free diet did not reach the levels of paralysis or rotarod impairment observed in littermate conditional α2-Na/K ATPase knockout mice on control diet (Supplementary Fig. 12e, f). Interestingly, although the serine- and glycine-free diet reversed the phenotype of transient motor paralysis in conditional α2-Na/K ATPase knockout mice, the survival of these mice was only modestly, albeit significantly, improved compared to conditional knockout mice on control diet (Fig. 6f). In control experiments, wild-type mice on either the control or serine- and glycine-free diet had no paralysis or impairment on rotarod, though the serine- and glycine-free diet led to some weight loss in control mice (Supplementary Fig. 12g–i). In other analyses, conditional α2-Na/K ATPase knockout mice fed the serine- and glycine-free diet that were switched to control diet at P40 displayed episodic transient paralysis, and impaired rotarod performance at P50 and P60 compared to conditional α2-Na/K ATPase knockout mice maintained on the serine- and glycine-free diet (Supplementary Fig. 13). We conclude that lowering serine and glycine levels in the brain selectively reverses the phenotype of transient motor paralysis in conditional α2-Na/K ATPase mice, establishing the critical role of serine and glycine elevation in the pathogenesis of transient motor paralysis in these mice.

## Discussion

In this study, we have discovered a metabolic mechanism by which loss of α2-Na/K ATPase in astrocytes triggers transient motor paralysis and sCSD in mice. We have found that mice in which α2-Na/K ATPase is conditionally deleted in astrocytes display the unexpected phenotype of episodic transient motor paralysis. In functional imaging studies, conditional knockout of α2-Na/K ATPase triggers sCSD events, a hallmark of migraine aura. The sCSD events in conditional α2-Na/K ATPase knockout mice are associated with LVA events upon EEG monitoring, which in turn correlate with transient episodes of motor impairments in these mice. In mechanistic studies, transcriptomic and metabolomic analyses reveal that astrocytic knockout of α2-Na/K ATPase induces alterations of metabolic gene expression in astrocytes with associated elevation of the amino acids serine and glycine in the brain. Strikingly, feeding mice a diet free of serine and glycine reverses the phenotype of transient motor paralysis, establishing a mechanistic link between elevation of serine and glycine and transient motor paralysis. Our study defines a link between the ion pump α2-Na/K ATPase and amino acid metabolism in astrocytes in the brain, whose deregulation triggers episodic transient motor paralysis in mice, with potential implications for understanding episodic neurological disorders in the human brain.

We unexpectedly discovered that mice in which α2-Na/K ATPase is conditionally deleted in astrocytes display episodic transient motor paralysis. Mutations of α2-Na/K ATPase cause familial hemiplegic migraine. Heterozygous knock-in mice bearing patient-specific mutations of α2-Na/K ATPase have provided insights into α2-Na/K ATPase function, bolstering the conclusion that mutations of α2-Na/K ATPase in familial hemiplegic migraine lead to loss of α2-Na/K ATPase function. However, although heterozygous knock-in mice with patient-specific mutations in α2-Na/K ATPase exhibit increased susceptibility to experimentally iCSD events, these mice do not display spontaneous episodic neurological disturbances^26,28–30^. The absence of episodic motor phenotypes in the α2-Na/K ATPase heterozygous knock-in mice may reflect the low frequency of neurological disturbances in the disorder or differences in the mouse and human brain. Notably, heterozygous conditional α2-Na/K ATPase knockout mice do not exhibit spontaneous motor impairment. The unexpected finding of spontaneous, episodic transient motor paralysis in conditional α2-Na/K ATPase knockout mice raises the question of whether these events might be relevant to the transient neurological disturbances in familial hemiplegic migraine. The correlation of transient motor impairment in conditional α2-Na/K ATPase mice with sCSD events, using the EEG surrogate of LVA suggests these events may be related to hemiplegic migraine aura. However, the high frequency of transient motor impairment, as well as the additional phenotypes of weight loss and premature death in conditional α2-Na/K ATPase mice leave open the possibility that transient motor paralysis in these mice represents a distinct pathophysiological process.

The identification of sCSD events in conditional α2-Na/K ATPase knockout mice may advance our understanding of the pathophysiology of migraine aura, which is associated with CSD events^39,40^. Further mechanistic studies in conditional α2-Na/K ATPase knockout mice should prove useful to address the fundamental question of how disruption of α2-Na/K ATPase activity in all astrocytes leads to localized deregulation of brain function in a CSD. The evaluation of total hemoglobin changes and neuronal calcium imaging during sCSD in this study have demonstrated the spatial heterogeneity of sCSD events, when compared to iCSD events. Further technological advances in the future should permit intrinsic optical signaling and wide-field GCAMP imaging in freely behaving mice, leading to characterization of how these specific spatial properties of CSD may correlate with motor phenotypes.

The link between loss of α2-Na/K ATPase function, and elevation of serine and glycine raises the question as to how α2-Na/K ATPase controls the levels of these amino acids. Our transcriptomics and metabolomics data suggest a role for gene expression alterations in enzymes that regulate serine and glycine metabolism, and deregulation of mitochondrial function. In vitro studies have implicated α2-Na/K ATPase in calcium signaling due to its association with the sodium–calcium exchanger, and deregulation of calcium leads to impairments in mitochondrial function^11,20,22,45,48^. Cellular stressors including mitochondrial dysfunction, oxidative stress, hypoxia and growth signals induce serine biosynthesis via changes in expression of genes, such as Shmt2 and Mthfd2 (refs. ^46,49,50^), which we have identified as deregulated in astrocytes upon conditional α2-Na/K ATPase knockout. Future studies will be required to identify the role of specific transcription factors that might couple loss of α2-Na/K ATPase function with elevation of serine and glycine levels in the brain.

An important aim of future work will be to delineate how elevated serine and glycine levels in the brain trigger motor impairment. Elevated cortical serine and glycine may increase susceptibility of the brain to CSD. Because D-serine is a synaptic NMDA receptor co-agonist, elevations in serine and related molecules might contribute to hyperexcitability of cortex via an increase of glutamatergic neurotransmission^51^. Consistent with this possibility, excessive glutamatergic signaling has been reported in knock-in mice bearing patient-specific mutations of α2-Na/K ATPase^26,28,29^.

The identification of elevated serine and glycine as a critical metabolic mechanism specifically in the pathogenesis of transient motor impairment rather than weight loss or premature death of conditional α2-Na/K ATPase mice suggests that episodic transient motor paralysis and phenotypes of weight loss and premature death upon depletion of astrocytic α2-Na/K ATPase may have distinct pathophysiological underpinnings. These observations also suggest a potential avenue for the investigation of amino acid metabolism in patients with familial hemiplegic migraine caused by α2-Na/K ATPase mutations, which may help to answer the question of whether the motor phenotype in conditional α2-Na/K ATPase mice is relevant to familial hemiplegic migraine, and hence advance our understanding of the disorder.

## Methods

### Animals

All animal experiments were done according to protocols approved by the Animal Studies Committee of Washington University School of Medicine and in accordance with the National Institutes of Health guidelines. Mice were purchased and maintained under pathogen-free conditions. Mice were kept on a 12 h on, 12 h off light cycle with room temperature 20–26 °C and humidity 30–70%. *mGFAP-Cre* mice were obtained from The Jackson Laboratory (JAX Stock No. 024098)^52^. mGFAP-Cre mice were mated with Ai3 YFP reporter mice (from The Jackson Laboratory, JAX Stock No. 007903)^53^ to generate mGFAP-Cre EYFP mice. mGFAP-Cre;*α2-Na/K ATPase* ^*loxP/+*^ mice were mated with *α2-Na/K ATPase* ^*loxP/loxP*^ mice^54^ to generate conditional α2-Na/K ATPase knockout mice (cKO: mGFAP-Cre;*α2-Na/K ATPase* ^*loxP/loxP*^), conditional α2-Na/K ATPase heterozygous mice (cHet: mGFAP-Cre;*α2-Na/K ATPase* ^*loxP/+*^) and control mice (Ctrl: *α2-Na/K ATPase* ^*loxP/loxP*^ or *α2-Na/K ATPase* ^*loxP/+*^). Once paralysis in conditional α2-Na/K ATPase knockout mice was frequent, we placed water gel and food on the cage bottom. Mice were monitored by weight and were culled if they reached their humane end point: 70% of maximum weight (survival = time to humane end point or death, whichever comes first). For wide-field calcium imaging in neurons, we generated conditional α2-Na/K ATPase knockout mice with GCaMP6f expression under the *Thy1* driver (mGFAP-Cre;*α2-Na/K ATPase* ^*loxP/loxP*^*;Thy1-GCaMP6f)* and controls (*α2-Na/K ATPase* ^*loxP/loxP*^ or *α2-Na/K ATPase* ^*loxP/+*^*;Thy1-GCaMP6f*). *Thy1-GCaMP6f* mice were obtained from The Jackson Laboratory (JAX Stock No. 024276)^55^. For TRAP-Seq experiments, we generated conditional α2-Na/K ATPase knockout mice with *mGFAP-Cre*-driven expression of EGFP fused to ribosomal protein unit L10A (JAX Stock No. 024750)^56^. All strains were backcrossed to and maintained on the C57BL/6J (black 6, JAX Stock No. 000664) strain of mice.

### Antibodies

Antibodies to α2-Na/K ATPase (Immunoblot, 1:20,000, Millipore, AB9094), α1-Na/K ATPase (Immunoblot, 1:5000, Thermo Fisher, MA3-928), α3-Na/K ATPase (Immunoblot, 1:5000, Thermo Fisher, MA3-915), actin (Immunoblot, 1:500, Santa Cruz, SC8432), GFP (Immunofluorescence, 1:500, Abcam, AB13970), NeuN (Immunofluorescence, 1:500, Abcam, AB177487), GFAP (Immunofluorescence, 1:500, Millipore, MAB3402), S100β (Immunofluorescence, 1:500, Abcam, AB41548), Iba1 (Immunofluorescence, 1:500, Wako, 019-19741), cleaved caspase-3 (Immunofluorescence, 1:500, Cell Signaling D175), and Alexa Fluor-conjugated secondary antibodies (Immunofluorescence, 1:500, Invitrogen, A11039, A11004, A11001, and A10042) were purchased.

### Immunoblotting analyses

Immunoblotting analyses were performed using standard techniques as previously described^57^. In brief, the cerebral cortex or gastrocnemius muscle from sex-matched littermate mice was homogenized in 50 mM Tris buffer (pH 7.4), 100 mM NaCl, 1 mM EDTA, and a cocktail of protease inhibitors and DTT. Equal concentrations were solubilized in SDS-loading buffer using a sonicator to prevent oligomerization of Na/K ATPase protein. Lysates were then subject to immunoblotting with antibodies to α1, α2, or α3-Na/K ATPase with actin as a loading control.

Immunoblot quantification was performed in FIJI using standard techniques. Mean gray value in an ROI of standardized area was calculated for each band, which was normalized to the value from a surrounding background area. Then, the mean intensity for the band of interest was divided by mean intensity for the loading control band for each sample.

### Immunofluorescence and hematoxylin and eosin staining

For immunofluorescence studies, conditional α2-Na/K ATPase knockout and sex-matched littermate f/f or f/+ control mice were anesthetized, and perfused with heparinized PBS followed by 4% PFA. The brain was extracted, postfixed, sucrose embedded, cryoprotected in 70% OCT in 30% sucrose, frozen, and sectioned into 30 µM sections on a cryostat. Sections were blocked in blocking buffer (3% bovine serum albumin, 10% normal goat serum, and 0.4% Triton-X in 1× PBS), stained overnight with antibodies against GFP, NeuN, GFAP, S100*β*, Iba1, and cleaved caspase-3 followed by incubation in appropriate Alexa Fluor-conjugated secondary antibodies, and imaged on a Nikon A1Rsi scanning confocal microscope. Cell nuclei were labeled with the DNA dye Hoechst 33258. For positive control for cleaved caspase-3 staining, cerebral cortices from mouse pups (P7–P21) injected subcutaneously with 2.5 mL/kg 20% ethanol 26 and 24 h prior to perfusion were used as a positive control for apoptosis. For H/E staining, the brain was extracted and fixed overnight in 4% PFA, then fixed brains were paraffin embedded, sectioned, and stained with H/E via standard methods.

### RNA-Seq and RT-qPCR

These experiments were performed as described^58^ with modifications. In brief, RNA was extracted from flash-frozen cerebral cortex of sex-matched littermate mice using Trizol (Thermo Fisher) followed by RNeasy Mini Kit (Qiagen) according to the manufacturers’ instructions. Each sample of RNA was made from the cortex of one mouse. For sequencing, 5–10 µg of RNA was reverse-transcribed with oligo-dT priming, and the cDNA was sequenced on an Illumina HiSeq 2000 (Genome Technology Access Center, GTAC, and McDonnell Genome Institute, MGI, at Washington University). Read mapping was performed in Galaxy with RNA STAR using the MM10 reference genome, and gene differential expression was estimated with EdgeR in R. The Benjamini–Hochberg false discovery rate was calculated for all genes; fold change and *Z*-scores were calculated based on counts per million (CPM). GO was performed using PANTHER^59^, Biological Process (Slim) unless otherwise specified. For qRT-PCR, real-time PCR reactions were performed using Light cycler 480 SYBR Green 1 Master (Roche). The following mouse gene primers were used: α2-Na/K ATPase forward: TTCGTAGCATCGTGGTTGT, α2-Na/K ATPase reverse: CACCACGTGACCTTGAGTGG, Gapdh forward: TGCTGGTGCTGAGTATGTCG, Gapdh reverse: GCATGTCAGATCCACAACGG, Gfap forward: AACCGCATCACCATTCCTGT, Gfap reverse: TCCTTAATGACCTCGCCATCC, Vimentin forward: AGACCAGAGATGGACAGGTGA, Vimentin reverse: TTGCGCTCCTGAAAAACTGC, Osmr forward: GTGAAGGACCCAAAGCATGT, Osmr reverse: GCCTAATACCTGGTGCGTGT, Srgn forward: GCAAGGTTATCCTGCTCGGA, Srgn reverse: TGGGAGGGCCGATGTTATTG, Serping1 forward: ACAGCCCCCTCTGAATTCTT, Serping1 reverse: GGATGCTCTCCAAGTTGCTC, Ggta forward: GTGAACAGCATGAGGGGTTT, Ggta reverse: GTTTTGTTGCCTCTGGGTGT, Cd109 forward: CACAGTCGGGAGCCCTAAAG, Cd109 reverse: GCAGCGATTTCGATGTCCAC, Emp1 forward: GAGACACTGGCCAGAAAAGC, Emp1 reverse: TAAAAGGCAAGGGAATGCAC, Clcf1 forward: CTTCAATCCTCCTCGACTGG, Clcf1 reverse: TACGTCGGAGTTCAGCTGTG^43^.

All primers performed well with standard curves and efficiency/correlation values for each pair shown in Supplementary Fig. 14. Standard curves were generated and PCR efficiency/correlation coefficient for each qRT-PCR primer pair as follows. A five-point twofold dilution series of cDNA was used to construct the assay, with Ct values being calculated from the average of two technical replicates. Ct values were plotted against the concentration of cDNA on a log scale, and a line was fit to this graph (Microsoft Excel). The slope of the line was used to calculate PCR efficiency (efficiency = 10^(−1/slope)^ − 1) and the correlation coefficient was measured from the degree of fit.

### Behavioral analyses

For quantification of motor function, mice were subjected to an accelerating or constant velocity rotarod test. For accelerating rotarod, mice were placed on a rod which accelerated from 3 to 20 r.p.m. over 180 s. For constant velocity rotarod, mice were placed on a rod which maintained 3 r.p.m. for 180 s. In both cases, mice were trained on day 1, prior to testing, by testing them on a stationary rod, and then at a constant 3 r.p.m. Mice passed training after staying on the stationary and then the 3 r.p.m. rod for a total of 60 s over three trials. Mice were tested by measuring their latency to fall for three trials, with an intertrial interval of ~10 min, and then averaging the three trials. For paralysis quantification, mice underwent video monitoring for 4 continuous hours for the specified timepoints until experimental end point or humane end point, whichever came first. Behavior was then scored, noting exact times when paralysis began or ended (paralysis is defined as lying on side or stomach and unable to right self within 60 s).

### TUNEL staining

For tissue staining, cortices were fresh frozen in embedding solution (70% tissue OCT + 30% sucrose) and cut into 15 µm sagittal sections. Sections were fixed by 4% paraformaldehyde for 20 min at room temperature, permeabilized by 0.1 M citrate buffer (pH 6.0) at 80 °C, treated with DNase working solution (10 µL 1000 U recombinant DNase + 900 µL DNase Tris buffer) for the positive control slice and tunnel labeling with 45 µL label solution together, with 5 µL enzyme solution for 1 h at room temperature in the dark. Sections were counterstained with Hoechst and mounted with Fluoromount before being visualized (In Situ Cell Death Detection Kit, Fluorescein, Sigma-Aldrich).

### Intrinsic optical signal imaging, wide-field calcium imaging, and EEG recording

Mice were put under general isofluorane anesthesia, while an anterior–posterior incision on the dorsal cortical surface was made to clear away the scalp and periosteal membranes. Animals undergoing craniotomy had a 1 mm diameter circle removed from the right posterior skull and were imaged immediately thereafter. Animals prepared for longitudinal imaging were implanted with stainless steel EEG bone screws and transparent cortical plexiglass windows as described^41^. Specifically, a unilateral right sided EEG screw was implanted −1 mm posterior and 5 mm lateral to bregma with a reference EEG screw in the cerebellum. The plexiglass window was fixed to the intact skull with translucent dental cement (C&B-Metabond, Parkell Inc, Edgewood, NY, USA), covering the cortical surface spanning from the olfactory bulbs to the superior colliculus. Mice were allowed 1 week to recover after surgery. EEG screws were attached to an amplifier and signal was collected at 10,000 Hz (Power Lab EEG Amplifier, AD Instruments, New Zealand) concurrent with iOS/fluorescence imaging. The mean EEG trace was subtracted from the signal and the result was plotted with the accompanying iOS/fluorescence data.

For iOS/fluorescence image acquisition, mice were suspended in a felt pouch or stereotaxic frame with the head fixed under the imaging apparatus. Sequential illumination of the cortex was provided by 454, 523, 595, and 640 nm LEDs (523, 595, and 640 nm used for iOS imaging; 454 nm used for GCaMP6f excitation); 523, 595, and 640 nm reflectance, as well as GCaMP6f fluorescence was recorded with a high-powered, cooled, frame-transfer EMCCD camera (Andor) through an 85 mm f/1.4 camera lens at 16.81 Hz per LED channel. A 515 nm longpass filter was used to filter out GCaMP6f excitation light.

As described elsewhere^60^, with modifications briefly summarized here, the iOS/GCaMP6f data was binned to 128 × 128 pixels at 78 μm^2^ per pixel (see “Code availability” statement below for github link to the code described here). Data were detrended and the mean trace for each channel was subtracted from the time series data to remove ambient light levels that may be present. Data were resampled to 1 Hz and a binary brain mask representing brain regions is applied, and a spatial and temporal detrending algorithm is used to correct for variance in light levels across pixels or time. The binary mask and data were then affine transformed to common atlas space. Data were mean normalized and the 523, 595, and 640 nm channels are translated into oxygenated and deoxygenated hemoglobin using the modified Beer–Lambert law. GCaMP6f fluorescence was corrected for hemodynamic fluctuations using a ratiometric approach using the 523 nm reflectance data. All data were smoothed with a 5 × 5 pixel Gaussian filter, and the global signal was determined as the average signal from all pixels and regressed from the data to control for global sources of variance.

For iCSD monitoring, mice were maintained under inhaled isoflurane anesthesia for the duration of imaging. Experimenters were blinded to the genotype of the mice. Mice underwent 10 min of baseline imaging, followed by three trials in succession of a dural pinprick followed by 20 min of imaging. Trials in which a CSD did not immediately follow the dural pinprick were discarded from further analysis. For sCSD monitoring, mice underwent surgery at least 7 days prior to the first imaging session. Mice were imaged for 90 min during each of the following age intervals: P27–P30, P31–P34, P35–P38, P39–P42, P43–P46, P47–P50, P51–P54, P55–P58, and P59–P62.

To calculate speed for CSDs, we did the following. For iCSD, since predominate direction of spread was in the posterior to anterior direction, signal was converted to a position-time plot by averaging the signal in the affected hemisphere for each *y* pixel location and plotting that over time. A linear regression was then used to fit a line to the peak of the signal. These lines are shown in Supplementary Figs. 5 and 6 for every data point included in the analysis. The slope of these lines was used to estimate CSD speed by converting it from pixels per seconds to mm/min using our measured 11 pixels/mm. To calculate EEG speed, we calculated the lag in minutes between the two waveforms using a cross correlation and divided the distance between electrodes in mm by that time. For sCSD events, since the CSDs could arise from anywhere and spread any direction, we first viewed videos and qualitatively determined the hemisphere of the CSD and whether it spread in the *x*-axis, *y*-axis, or both. We then created position-time plots for the axis or axes of spread in the appropriate hemisphere, and calculated speed as outlined for iCSD events. The axis and hemisphere of spread are presented in a table in Supplementary Fig. 8, as are all regression lines and speeds.

### Continuous EEG and video recording

Conditional α2-Na/K ATPase knockout male and female mice were obtained for EEG analysis (*n* = 7; 2 male, 5 female α2-Na/K ATPase conditional knockout mice; *n* = 6; 3 male/3 female control). The control EEG appeared normal after the first week of monitoring. Mice received surgery for placement of EEG electrodes at P21. Custom wire EEG electrode sets were constructed using five Teflon-coated stainless steel wires (76 µm bare diameter) soldered to an electronic pin header and a micro screw. The soldered contacts were covered with dental cement and the electrode set sterilized for implantation. Mice were placed under 3–4% isoflurane on a stereotaxic frame with a heating pad set to 36.5 °C until pedal withdraw reflex ceased. The skin was prepared with betadine and alcohol wipes with isoflurane maintained at 1–1.5% for the remainder of the procedure. After a midline vertical incision to expose the skull, forceps, and 3% hydrogen peroxide were used to remove any connective tissue and dry the skull for electrode placement. Burr holes for the frontal reference electrodes were made (anterior +0.5 mm, lateral ±0.5 mm; bregma), using a micro drill with a 0.9 mm tip and screws were secured in the skull. Two bilateral active recording electrodes were placed over the parietal cortex (posterior −2.5 mm, lateral ±1.5; bregma) and a ground screw secured over the cerebellum (posterior −6.2 mm, lateral ±0.5; bregma), using the same technique as the reference electrode. The exposed skull, screws, and all wires were covered in a layer of dental cement (SNAP, Parkell) with the pin header secured to the head for subsequent recording. The skin was sutured around the exposed dental cement/pin header and tissue glue (Vetbond, 3 M) used to close the remainder of the incision. Mice received Buprenorphine (0.1 mg/kg) and recovered in a warmed chamber for 2 h then placed in individual recording cages.

Groups of four mice in individual caging recovered from surgery for 48 h prior to connection with a custom flexible cable attached to the exposed pin header for recording. Due to their small size, EEG tethering significantly limits mobility of α2-Na/K ATPase conditional knockout mice at 3 weeks of age. Thus, 3-week-old mice were monitored for an initial 48 h recording period then untethered for the remainder of the week. Starting at 4 weeks of age, the mice were tethered for continuous (24/7) video-EEG monitoring for the remainder of the experiment. Bilateral cortical EEG signals were acquired using a referential montage using Stellate acquisition software and amplifiers. Signals were amplified at 10,000× with high-pass (0.5 Hz) and low-pass (100 Hz) filters applied. EEG signals were digitized at 250 Hz and time-locked video-EEG collected continuously for 3–4 weeks.

Continuous 24-h video-EEG of mice was manually screened for seizures and EEG abnormalities. Conditional α2-Na/K ATPase knockout mice displayed LVA on EEG, which consisted of abrupt voltage suppression of the normal EEG background usually containing ~3 Hz breathing artifact followed by burst suppression activity gradually returning to normal. Quantitatively, LVA events were defined by EEG background with a >75% reduction in total power within 15 s, lasting <10 min in duration. Defined periods of abnormal EEG were annotated and time-locked video observed to determine a correlation between behavior and EEG events. Behavior was scored in a blinded fashion; during an LVA event, the behavior of all mice in that cohort was scored without knowing which mouse had the EEG abnormality. Behavior in the following categories was noted: sleeping, eating, normal ambulation, abnormal ambulation (ataxic gait), abnormal stationary posture (rearing behavior), and paralysis (lying on stomach or side and unable to right self). Once all behaviors were recorded for each EEG abnormality, the reviewer used a random number generator to select one control mouse from the mice not exhibiting an EEG abnormality and was unblinded to quantify the behavior of mice exhibiting EEG abnormalities.

### TRAP-Seq analyses

TRAP was performed on the cortex from three replicates of control and conditional knockout mice at age P24 modified from protocols as described^61^. Briefly, the bilateral cortices from each mouse was dissected out and, separately for each mouse, homogenized in 2 mL homogenization buffer (20 mM Hepes-KOH, 150 mM Kcl, and 10 mM Mgcl2) with RNase inhibitor, and 10% NP40 and 300 mM DHPC (Avanti Polar Lipids) was added to the supernatant. After spinning down, 10% of the supernatant was used as for the input fraction with the rest being subjected to immunoprecipitation (IP). GFP-ribosome positive ribosomes were pulled down by incubation with affinity antibody matrix (19C8 and 19F7, Memorial Sloan-Kettering Monoclonal antibody facility) at 4 °C overnight. RNA was collected by Qiagen miRNA kit. RNA quality and concentration were assessed using high sensitivity RNA ChiP on the Agilent BioAnalyzer. Intact RNA (RIN > 8, ~100 ng per sample) was used for the library using NEBNext UltraRNA library preparation kit (E7530) as described^62,63^. RNA was purified by oligdT beads and cDNA was synthesized by reverse transcriptase. After DNA fragmentation, end repair, adaptor ligation and PCR amplification, and libraries were used for Illumina deep sequencing. Sequencing of the RNA was performed as described above. Sequencing was performed on 12 samples: input from each cortical samples of three control mice, IP from cortical samples of three control mice, input from cortical samples of three conditional knockout mice and IP from cortical samples of three conditional knockout mice.

### Targeted metabolomics (liquid chromatography–mass spectrometry)

Metabolites from powderized flash-frozen brain tissue were extracted in ice-cold 80% methanol. Subsequently, extract from the equivalent of 1 mg tissue was dried down in a room temperature speed-vac for metabolomics analysis. Metabolites were resolved using reverse phase ion-paired chromatography on an Agilent 1290 Infinity II Series LC and detected by MRM in negative ion mode on an Agilent 6470 series triple quadrupole mass spectrometer. The liquid chromatography–tandem mass spectrometry (LC–MS/MS) targeted MRM method used for metabolomics analysis was developed and implemented by Agilent Technologies.

### High-performance liquid chromatography

Flash-frozen brain tissue was dissociated in 50 µL perchloric acid per mg of tissue (wet) and flash frozen. Derivatization, detection, and quantification of D-serine and L-serine were then performed as described^64^. Briefly, 2.5 µL of sample were derivatized in 2.5 µL of aqueous phase with an excess (5 µL) of OPA/MAC mix (0.02 g o-Phthaldialdehyde in 1 mL methanol + 0.01g *N*-acetyl-L-cysteine + 9 mL OPA diluent) for 2 min, followed by treatment with 2 µL of 1 M acetic acid. Chiral separation of amino acids in the derivatized samples was achieved via HPLC on a modular Shimadzu system (C10 Accucore column, 150 × 2.1 mm, 2.6 µm pores) at 40 °C, at a flow rate 0.2 mL/min using a non-isocratic method (0–6 min 3% organic, 6–10 min 20% organic, 10–14 min 80% organic). Injection volume was 1 µL. The organic phase consisted of 100% methanol and the aqueous phase of 50 mM phosphate buffer (pH = 4.34). For quantification, standards of D-serine and L-serine were prepared in aqueous mobile phase and treated similarly to the samples. The area under the D-serine and L-serine peaks was resolved using LabSolution software to obtain D- and L-serine concentration in the samples relative to their respective standards.

### Gas chromatography–mass spectrometry

Mice were perfused with 25 mL cold heparinized PBS and tissues flash frozen. Metabolites were extracted from tissue homogenized in 10 µL/mg wet weight of 65% EtOH by sonicating for 30 s at 50% amplitude, spun 10 min at 6000 × *g*. Supernatants were pooled, labeled standards were added, and then the solution was lyophilized using a speed-vac. Derivatization was carried out by adding 75 ul MSTFA *(N*-methyl-*N* trimethylsilyltrifluoroacetamide)/CH_3_CN(1:3) and heating for 30 min at 70 °C.

Derivatized samples were analyzed on an Agilent 7890A gas chromatograph interfaced to an Agilent 5975C mass spectrometer. The GC column used for the study was a HP-5MS (30 m, 0.25 mm i.d., 0.25 µm film coating P.J. Cobert St. Louis, MO). A linear temperature gradient was used. The initial temperature of 80° was held for 2 min and increased to 300 at 10°/min. The temperature was held at 300 °C for 6 min. The samples were run by electron ionization and the source temperature, electron energy and emission current were 250 °C, 70 eV, and 300 µA, respectively. The injector and transfer line temperatures were 250 °C.

Glycine quantitation was carried out by monitoring the ions at *m*/*z* 276 for glycine and 278 for glycine-d2. Serine quantitation was carried out by monitoring the ions at *m*/*z* 306 for serine and 309 for serine-d3.

### Transmission electron microscopy

P24 mice were pericardially perfused for 2 min with 37C Ringer’s solution followed by 5 min with 37C TEM fixative (0.15 M cacodylatte buffer Ted Pella pH 7.4 containing 2.5% glutaraldehyde-EMS and 2% paraformaldehyde-EMS with 2 mM calcium chloride), then tissue fixed at least overnight in 4 °C TEM fixative. Sections were made with a Leica EM UC7 Ultramicrotome. Images were taken from L2/3 of motor cortex using a JEOL JEM-1400 120 kV TEM. Astrocytes were recognized morphologically focusing on perivascular astrocytes for ease of morphological identification. Abnormal vs. normal astrocytic mitochondria were judged by an experienced, blinded observer (see Supplementary Fig. 11a for examples) using the criteria as follows. Astrocytes were identified at low magnification (3000×) based on their classic EM morphology (nucleus and cytoplasm), and presence of bundles of cytoplasmic cytoskeletal intermediate filaments. They were then observed at higher magnification (8000×). Both cross sectioned and longitudinally sectioned mitochondria were studied. Identification of abnormal mitochondria were based on the following criteria—overall morphology, intactness of the mitochondrial double layer membrane, distortions or abnormal swellings of mitochondria cristae, localized changes in mitochondrial matrix, such as vacuolization, compaction of membranes and extreme density. Mitochondrial morphology in the neighboring neuropil (blood vessels, dendrites, axons, axonal terminals, etc.) was compared as reference.

### Serine- and glycine-free diet

Modified diets were purchased from LabDiet. Four modified diets were used: Ctrl (5BQS) and serine- and glycine-free diet (5BQT) as previously described^65^, and serine-free (5WNT) and glycine-free (5WNU). The serine- and glycine-free and Ctrl diets are the same except that all serine and glycine is replaced by other amino acids in the same ratios such that the total protein percentage is comparable. Serine-free and glycine-free are the same as serine- and glycine-free, except that serine-free has glycine added back in and glycine-free has serine added back in, again, with same percentage total protein. Animals were weaned directly onto the modified diets at 17 days of age and stayed on the modified diet for their entire life. All animals were given free access to water. A subset of animals was weighed daily starting at 17 days of age, and/or began behavioral testing at 30 days of age, including daily constant velocity rotarod testing and every other day paralysis monitoring. These experiments continued for the lifespan of the mice. All experiments, including weights, paralysis monitoring, and rotarod, were conducted with the experimenter blinded to the diet and the genotype of the mice. For studies on the effect of the reversal of the diet, mice were treated as above with one exception: one cohort of mice was switched from the serine- and glycine-free diet to the control diet at P40.

### Statistical analysis

Statistical analyses were done using GraphPad Prism software. Bar graphs are presented as the mean ± s.e.m. For experiments in which only two groups were analyzed, the two-tailed unpaired *t* test was used. No statistical methods were used to predetermine sample sizes, but our sample sizes are similar to those generally employed in the field. Data distribution was assumed to be normal, but this was not formally tested. Details on statistical results including exact *P* values are provided in the Supplementary statistics data file (Supplementary Data 2) or in the figure legend if space allows.

### Reporting summary

Further information on research design is available in the Nature Research Reporting Summary linked to this article.

## Supplementary information

Supplementary Information

Description of Additional Supplementary Files

Supplementary Movie 1

Supplementary Movie 2

Supplementary Movie 3

Supplementary Movie 4

Supplementary Movie 5

Supplementary Movie 6

Supplementary Movie 7

Supplementary Movie 8

Supplementary Movie 9

Supplementary Movie 10

Supplementary Movie 11

Supplementary Data 1

Supplementary Data 2

Reporting Summary

## Data Availability

All sequencing data is deposited into GEO with accession code GSE145012. Differentially expressed genes and metabolomics source data are provided in Supplementary Data 1. Statistical details for all statistical tests including exact *P* values included in Supplementary Data 2. All data supporting our findings can be found within the article and its Supplementary Information.  Source data are provided with this paper.

## Source data

Source Data

## Code availability

Custom code used for intrinsic optical signal (iOS) imaging, wide-field calcium imaging and EEG acquisition, processing, and analysis is provided on github (https://github.com/brierl/Nature_Comm_CSD).
