# Supplementary material for: Astrocyte deletion of α2-Na/K ATPase triggers episodic motor paralysis in mice via a metabolic pathway
Source: Nat Commun. 2020 Dec 2;11:6164. doi: 10.1038/s41467-020-19915-2 (PMC7710756; doi:10.1038/s41467-020-19915-2)
Supplement: Supplementary file 1 — Supplementary Information [file 41467_2020_19915_MOESM1_ESM.pdf]

**Supplementary Information**

# Astrocyte Deletion of α2-Na/K ATPase Triggers Episodic Motor Paralysis in Mice via a Metabolic Pathway

Sarah E. Smith[1,2], Xiaoying Chen[1], Lindsey M. Brier[2,3], Jonathan R. Bumstead[3,4], Nicholas R. Rensing[5], Alison E. Ringel[6], Haewon Shin[1], Anna Oldenborg[1], Jan R. Crowley[7], Annie R. Bice[3], Krikor Dikranian[1], Joseph E. Ippolito[3], Marcia C. Haigis[6], Thomas Papouin[1], Guoyan Zhao[1], Michael Wong[5], Joseph P. Culver[3,4,8], Azad Bonni[1,*]

[1]Department of Neuroscience, Washington University School of Medicine, St. Louis, MO 63110, USA
[2]MD-PhD Program, Washington University School of Medicine, St. Louis, MO 63110, USA
[3]Department of Radiology, Washington University School of Medicine, St. Louis, MO 63110, USA
[4]Department of Biomedical Engineering, Washington University in St. Louis, St. Louis, MO 63105, USA
[5]Department of Neurology, Washington University School of Medicine, St. Louis, MO 63110, USA
[6]Department of Cell Biology, Blavatnik Institute, Harvard Medical School, Boston, MA 02115, USA
[7]Department of Internal Medicine, Washington University School of Medicine, St. Louis, MO 63110, USA
[8]Department of Physics, Washington University in St. Louis, St. Louis, MO 63105, USA
*Correspondence: bonni@wustl.edu

**This PDF file includes:**
Supplementary Figures 1-15

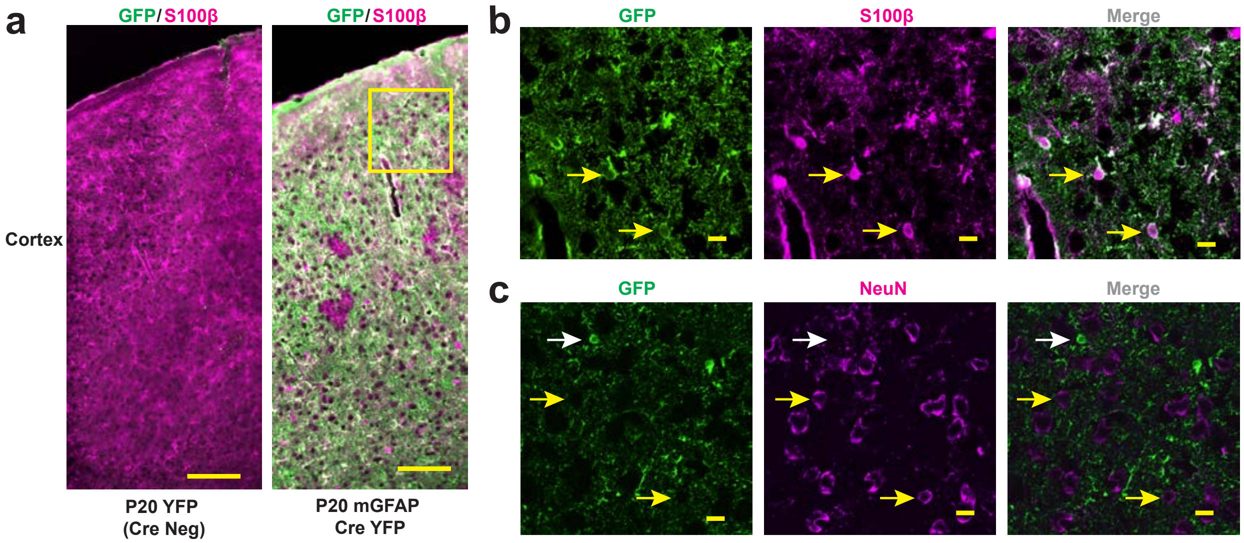


**Supplementary Fig 1 The *mGFAP-Cre* driver is expressed predominantly in astrocytes within the cortex in the postnatal mouse brain.**

**a-b.** The cerebral cortex from P20 *mGFAP-Cre YFP* and Cre-negative *YFP* control mice were subjected to immunofluorescence analyses using antibodies to GFP (green) and the astrocyte marker S100*β* (magenta). Scale bar represents 100µm in left larger image (**a**) and 10µm in inset (**b**). Yellow arrows in (**b**) indicate 2 example astrocytes with expression of GFP and S100*β* in a colocalized fashion in the cell body (n=2)

**c.** The cerebral cortex from P20 *mGFAP-Cre YFP* mice were subjected to immunofluorescence analyses using antibodies to GFP (green) and the neuron marker NeuN (magenta). Scale bar represents 10µm. Yellow arrows indicate 2 example neurons, which express NeuN but no GFP. White arrow indicates an example astrocyte expressing GFP but no NeuN (n=2).

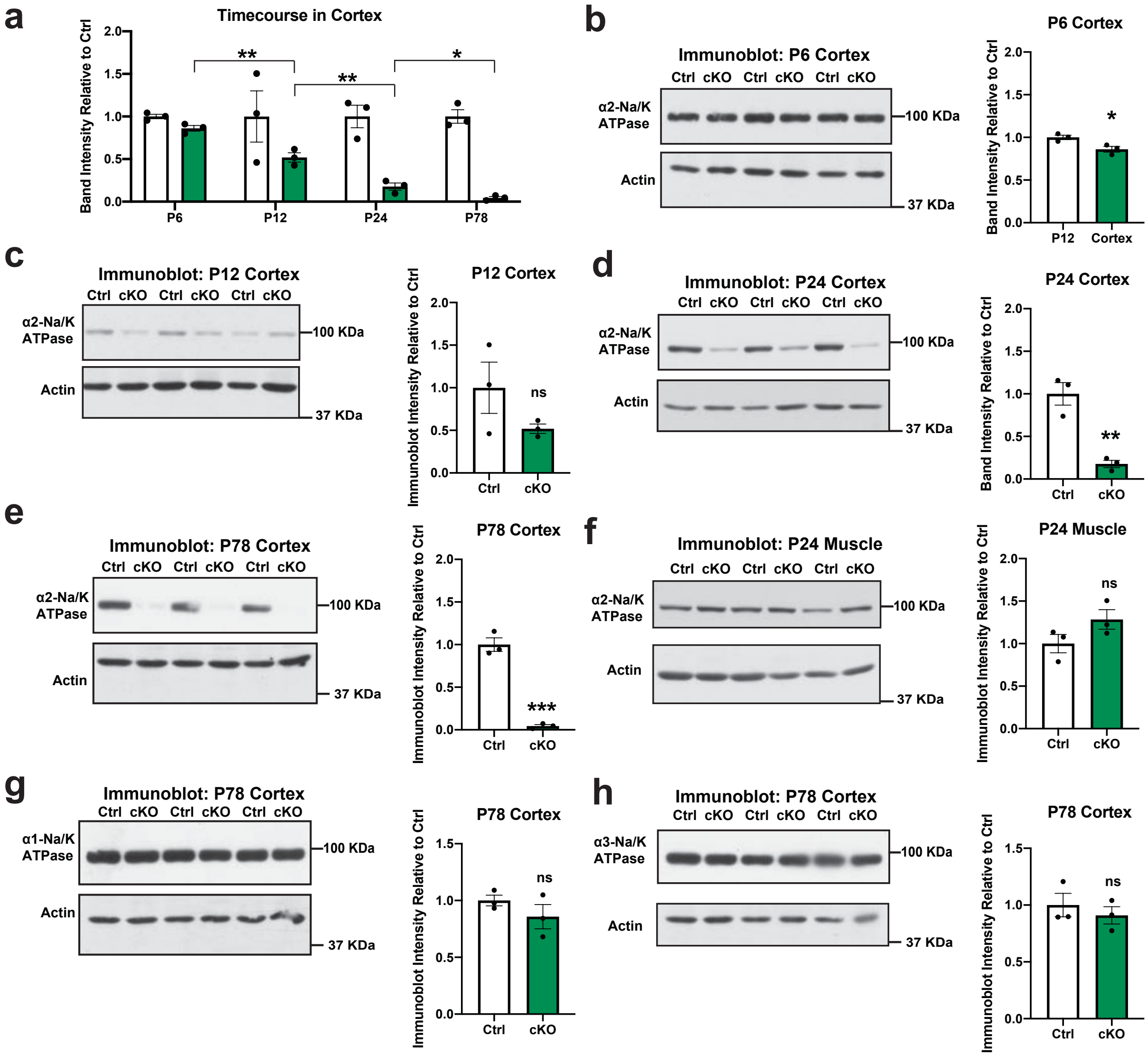


**Supplementary Fig 2 Conditional astrocytic α2-Na/K ATPase knockout mice exhibit robust knockdown of α2-Na/K ATPase protein levels by P24 in cortex.**

**a-h.** Lysates of the cerebral cortex (**a-e, g-h**) or gastrocnemius muscle (**f**) from P6 (**a, b**), P12 (**a, c**), P24 (**a, d, f**) or P78 (**a, e, g-h**) mice harboring the floxed α2-Na/K ATPase gene (f/f or f/+, Ctrl) and sex-matched littermate f/f α2-Na/K ATPase mice crossed with mice expressing the recombinase Cre under the control of the mGFAP gene promoter (conditional α2-Na/K ATPase knockout, cKO) were immunoblotted with α2-Na/K ATPase (**a-f**), α1-Na/K ATPase (**g**) or α3-

Na/K ATPase (**h**) and actin antibodies, the latter serving as loading control. Blots are pictured on the left of each subplot, quantification of that blot is on the right, except in (**a**) which presents the quantification of all cortical cKO blots together for statistical comparison. Data are presented as mean ± s.e.m. *$P<0.05$, **$P<0.01$, ***$P<0.001$. (Two-tailed *t* test, n=3 mice per group, a: p=0.006 P6-P12, p=0.0079 P12-P24, p=0.044 P24-P78, b: p=0.030, c: p=0.19, d: p=0.0041, e: 0.15, f: p=0.0003, g: p=0.29, h: p=0.52). All full blots are presented in Supplementary Fig 15. Sex of pairs of mice were, in order from left to right, female female male (**b**), female female female (**c**), and male female female (**d-h**).

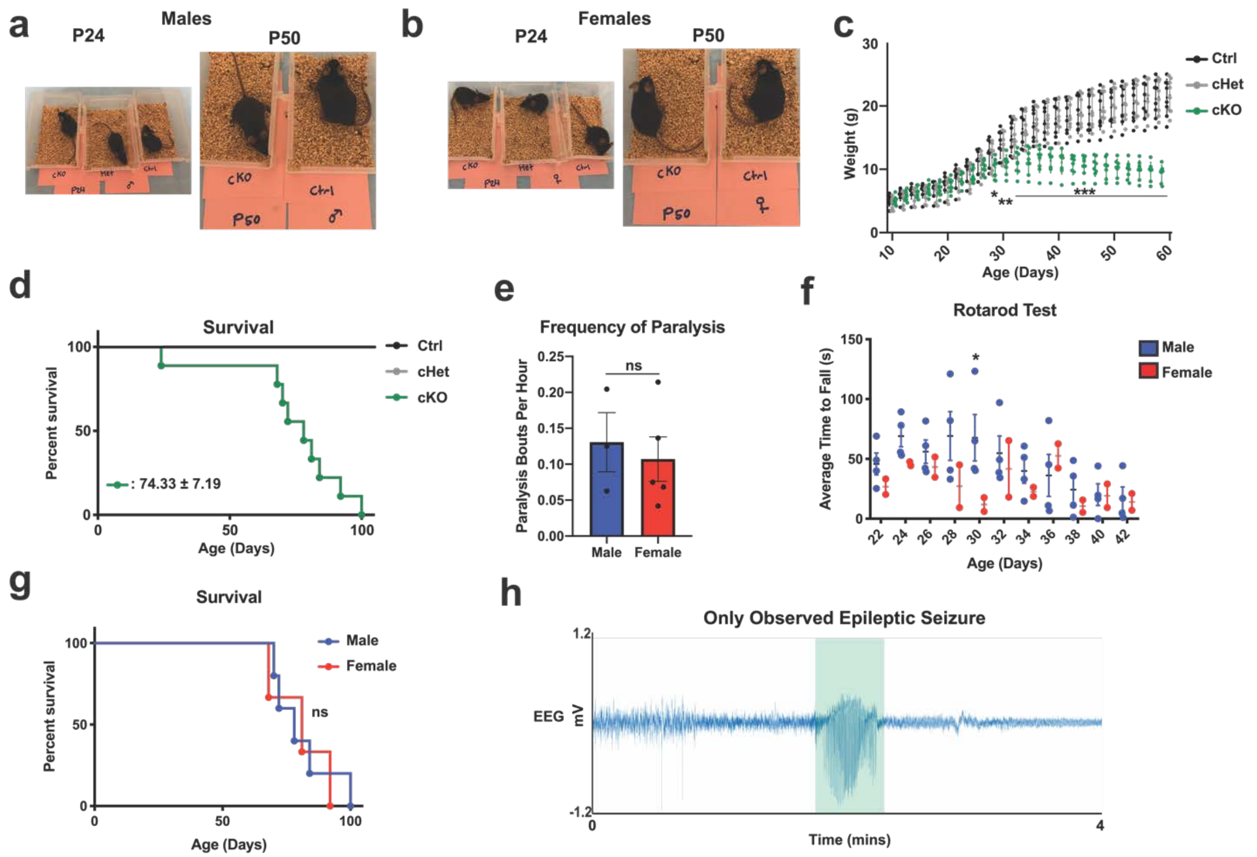


**Supplementary Fig 3 Conditional astrocytic α2-Na/K ATPase knockout mice of both sexes are viable into adulthood but exhibit small size and premature death.**

**a-b.** Photographic images of, from left to right in the P24 image, a sex-matched male littermate set of conditional α2-Na/K ATPase knockout mice (cKO), conditional α2-Na/K ATPase heterozygous mice (cHet) and f/f or f/+ control (Ctrl) mice, and just cKO and Ctrl in the P50 image for clarity. Smaller photo on the left is from when the mice were P24, larger photo on the right is when the mice were P50. Both male (**a**) and female (**b**) littermates are shown.

**c.** Sex-matched littermate cKO, Ctrl and cHet mice were weighed every other day from 10 days to 60 days of age. cKO mice were not significantly different than Ctrl mice from 10 days to 26 days (P>0.05), but were significantly lower in weight from 28 days to 60 days. Data are

presented as mean ± s.e.m. *$P<0.05$, **$P<0.01$, ***$P<0.001$ (Two-way ANOVA followed by Fisher's LSD *post hoc* test, n=8 mice per group).

**d.** Sex-matched littermate cKO, Ctrl and cHet mice were monitored daily for survival. Data are presented as mean ± s.e.m. ns $P>0.05$ (n=9 mice per group).

**e.** Male and female cKO mice were subjected to video monitoring and onsets and offsets of paralysis bouts were recorded. A paralysis bout was defined as a time period in which mice were on their abdomen or side and unable to right themselves for at least 60 seconds. Mice were recorded for a 4-hour session every 5 days from P25 until P75 or euthanasia. Quantification of video monitoring from cKO and Ctrl mice showing number of paralysis bouts. Data are presented as mean ± s.e.m. Data also shown in Fig. 1d without separating by sex. ns $P>0.05$ (Two-tailed *t* test, n=3 male, 5 female mice, p=0.66).

**f.** Male and female cKO mice were subjected to an accelerating rotarod test every other day from 22 days to 42 days of age. Data also shown in Fig. 1f without separating by sex. (n=4 male mice, 2 female mice).

**g.** Male and female cKO mice were monitored daily for survival. ns $P>0.05$. (Log-Rank (Mantel-Cox) test, n=5 male, 3 female mice).

**h.** Throughout all EEG recording for all mice, despite recording from both cortices continuously for the lifespan of the animals, only one epileptic seizure was captured (in shaded box). It occurred in a cKO mouse at P39 and was accompanied by hyperactivity and myoclonus, not paralysis or ataxia.

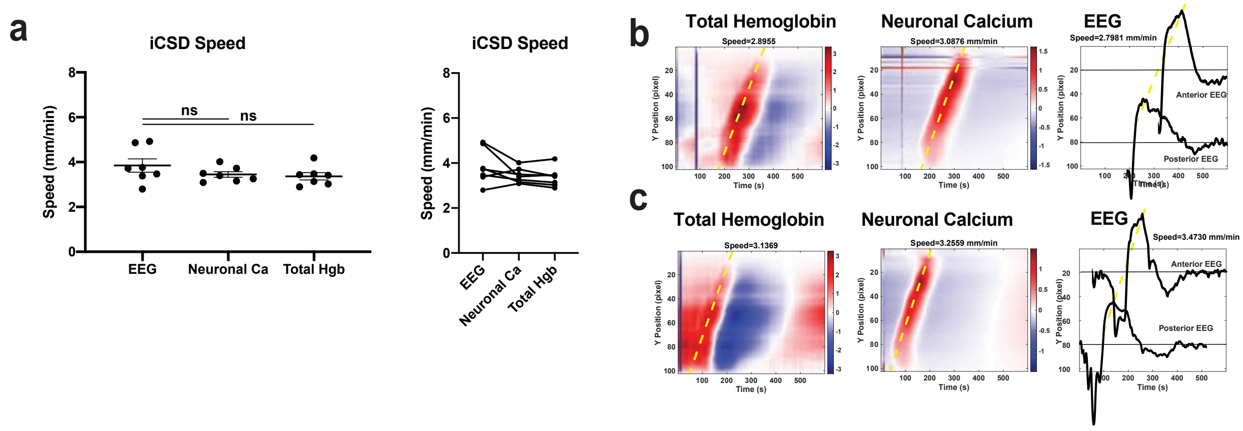


**Supplementary Fig 4 EEG validation of CSD speed measurements for Total Hemoglobin and Neuronal Calcium.**

**a-c.** Cortical spreading induced with dural pinprick proceeds at a similar speed as measured by two spaced EEG leads and via Neuronal calcium and Total Hemoglobin (**a**). Data are presented as mean ± s.e.m. ns $P>0.05$ (Two-tailed *t* test, n=7 iCSDs in 2 male and 2 female 12-15 month old mice, p=0.18 neuronal calcium and p=0.70 total hgb). Example iCSDs from a male (**b**) and female (**c**) control mouse. Left: average signal at each y point on the brain, for each point in time, is made into an image (position-Time plot) where the color of the image represents the signal, y axis represents the y-position on the brain and x axis represents time. Lines were fit to the peak of the position-time plot, as displayed by the yellow dotted line, the slopes of which were used to calculate the speed of the CSD in mm/min. Speed of each iCSD is shown above the position-time plot, which is the data shown in aggregated form in (**a**). Units of the scale is change in uM hemoglobin for total hemoglobin and change in fluorescence for neuronal calcium. Right: EEG data and the y-location that it corresponds to. The yellow dotted line represents the speed calculated for EEG.

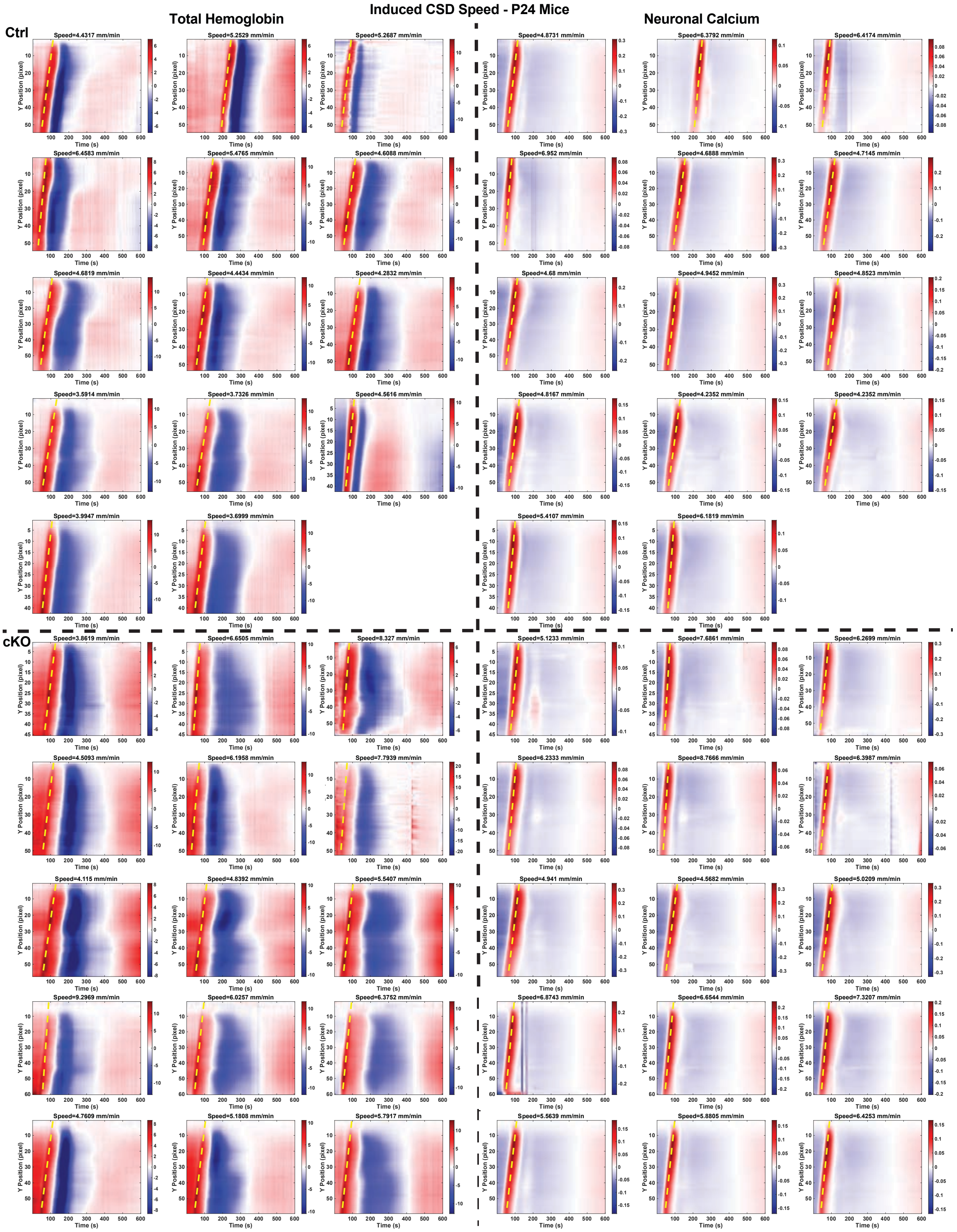

Induced CSD Speed - P24 Mice
Total Hemoglobin
Neuronal Calcium
Ctrl
Speed=4.4317 mm/min
Speed=5.2529 mm/min
Speed=5.2687 mm/min
Speed=4.8731 mm/min
Speed=6.3792 mm/min
Speed=6.4174 mm/min
Speed=6.4583 mm/min
Speed=5.4765 mm/min
Speed=4.6088 mm/min
Speed=6.952 mm/min
Speed=4.6888 mm/min
Speed=4.7145 mm/min
Speed=4.6819 mm/min
Speed=4.4434 mm/min
Speed=4.2832 mm/min
Speed=4.68 mm/min
Speed=4.9452 mm/min
Speed=4.8523 mm/min
Speed=3.5914 mm/min
Speed=3.7326 mm/min
Speed=4.5616 mm/min
Speed=4.8167 mm/min
Speed=4.2352 mm/min
Speed=4.2352 mm/min
Speed=3.9947 mm/min
Speed=3.6999 mm/min
Speed=5.4107 mm/min
Speed=6.1819 mm/min
cKO
Speed=3.8619 mm/min
Speed=6.6505 mm/min
Speed=8.327 mm/min
Speed=5.1233 mm/min
Speed=7.6861 mm/min
Speed=6.2699 mm/min
Speed=4.5093 mm/min
Speed=6.1958 mm/min
Speed=7.7939 mm/min
Speed=6.2333 mm/min
Speed=8.7666 mm/min
Speed=6.3987 mm/min
Speed=4.115 mm/min
Speed=4.8392 mm/min
Speed=5.5407 mm/min
Speed=4.941 mm/min
Speed=4.5682 mm/min
Speed=5.0209 mm/min
Speed=9.2969 mm/min
Speed=6.0257 mm/min
Speed=6.3752 mm/min
Speed=6.8743 mm/min
Speed=6.6544 mm/min
Speed=7.3207 mm/min
Speed=4.7609 mm/min
Speed=5.1808 mm/min
Speed=5.7917 mm/min
Speed=5.5639 mm/min
Speed=5.8805 mm/min
Speed=6.4253 mm/min
Y Position (pixel)
Time (s)

**Supplementary Fig 5 Induced cortical spreading depression is faster in conditional α2-Na/K ATPase knockout mice at P24.**

All included iCSDs from P24 α2-Na/K ATPase knockout (cKO) and sex-matched littermate f/f or f/+ controls (Ctrl) mice. The average signal at each y point on the brain, for each point in time, is made into an image (position-Time plot) where the color of the image represents the signal, y axis represents the y-position on the brain and x axis represents time. Lines were fit to the peak of the position-time plot, as displayed by the yellow dotted line, the slopes of which were used to calculate the speed of the CSD in mm/min. Speed of each iCSD is shown above the position-time plot, which is the data shown in aggregated form in Fig 2e. Ctrl mice are shown in the top half, cKO mice are shown in the bottom half, total hemoglobin position-time plots are shown in the left half and neuronal calcium position-time plots are shown in the right half. Units of the scale is change in uM hemoglobin for total hemoglobin and change in fluorescence for neuronal calcium.

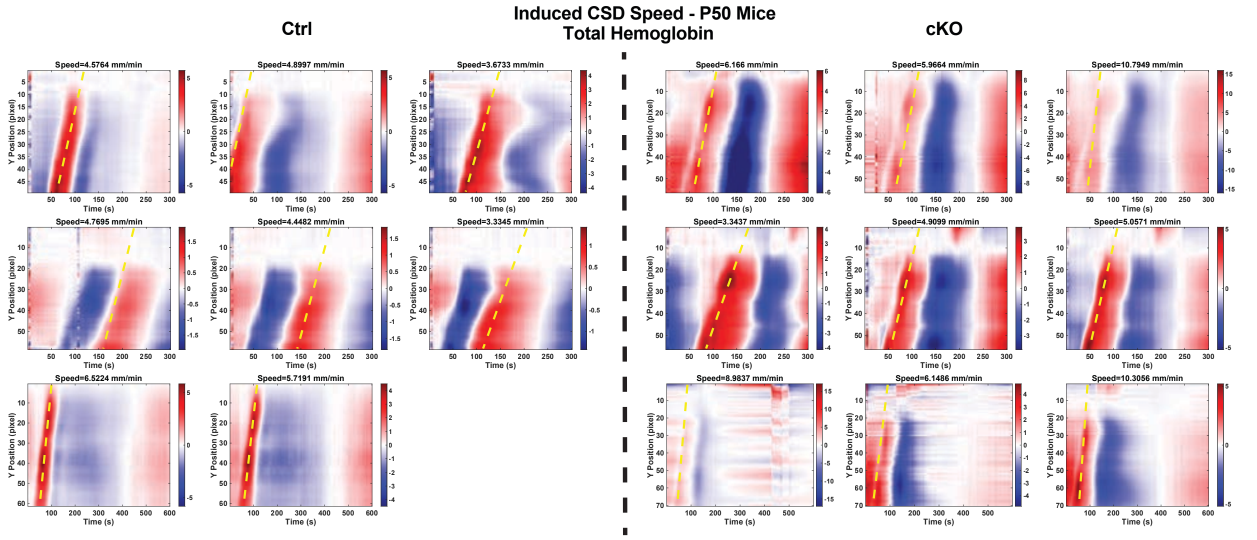


**Supplementary Fig 6 Induced cortical spreading depression is faster in conditional α2-Na/K ATPase knockout mice at P50.**

All included iCSDs from P50 α2-Na/K ATPase knockout (cKO) and sex-matched littermate f/f or f/+ controls (Ctrl) mice. The average signal at each y point on the brain, for each point in time, is made into an image (position-Time plot) where the color of the image represents the signal, y axis represents the y-position on the brain and x axis represents time. Lines were fit to the peak of the position-time plot, as displayed by the yellow dotted line, the slopes of which were used to calculate the speed of the CSD in mm/min. Speed of each iCSD is shown above the position-time plot, which is the data shown in aggregated form in Fig 2f. Ctrl mice are shown in the left half, cKO mice are shown in the right half. Units of the scale is change in uM hemoglobin.

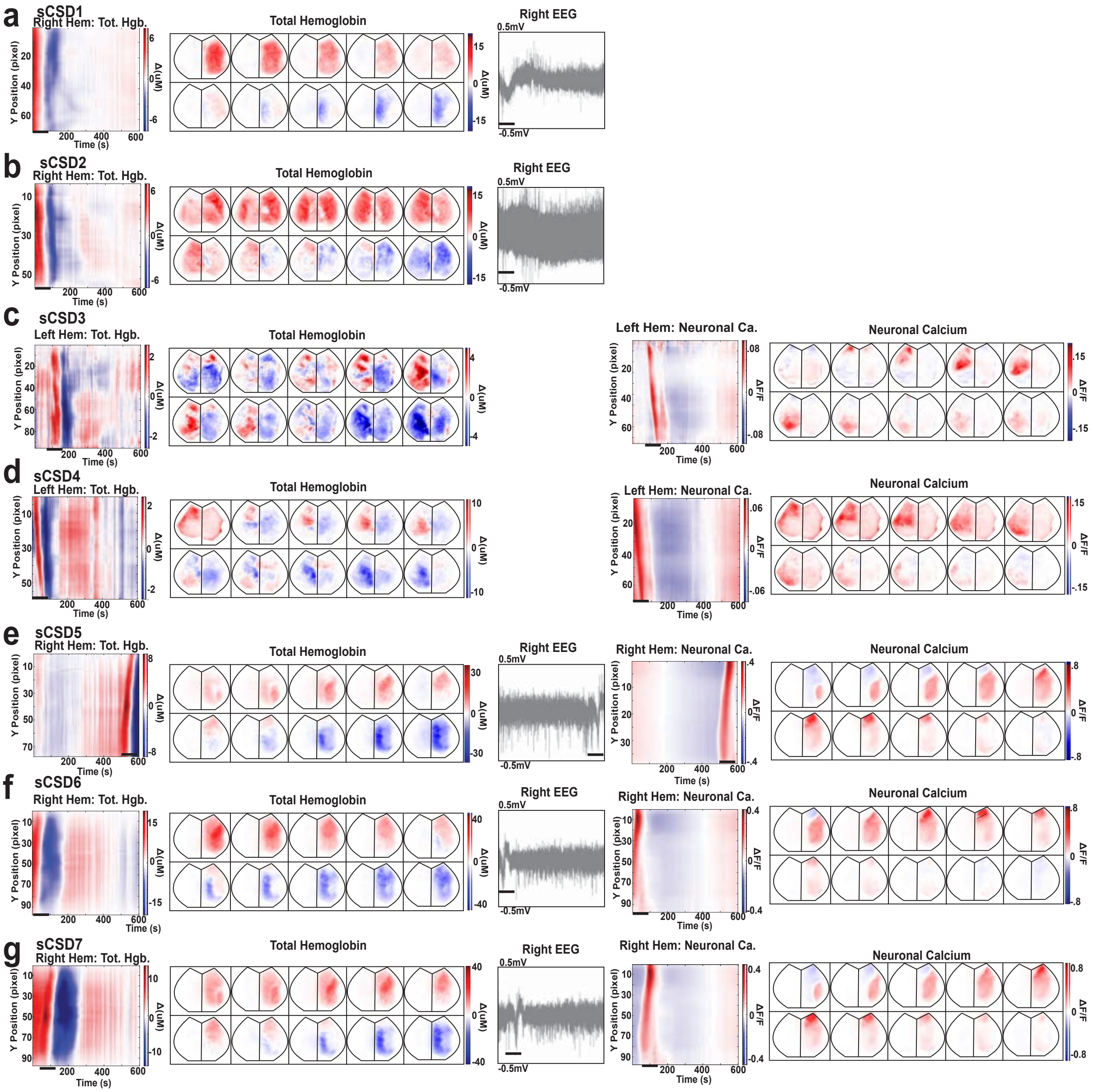

**Supplementary Fig 7 Low voltage activity occurs during spontaneous cortical spreading depressions.**

**a-g.** Low voltage activity occurs during all recorded hemispheric spontaneous cortical spreading depressions for which the sCSD occurred in the same hemisphere as the EEG lead (right). All spontaneous cortical spreading depression (sCSD) events were recorded from unanaesthetized conditional α2-Na/K ATPase knockout (cKO) mice. The CSD number corresponds to the table

in Fig 3c, which gives more information on each CSD. Time between still images is 10 seconds, and total span of still images corresponds to the black bars on the EEG and position-time plots. sCSD 1 **(a).** Still images start at 0 seconds. sCSD1 also depicted in Supplementary Movie 5. sCSD 2 **(b).** Still images start at 0 seconds. sCSD2 also depicted in Supplementary Movie 6. sCSD 3 **(c).** Still images start at 80 seconds. sCSD3 also depicted in Supplementary Movie 7. sCSD 4 **(d).** Still images start at 0 seconds. sCSD4 also depicted in Supplementary Movie 8. sCSD 5 **(e).** Still images start at 500 seconds. sCSD5 also depicted in Supplementary Movie 9. sCSD 6 **(f).** Still images start at 0 seconds. sCSD6 also depicted in Supplementary Movie 10. sCSD 7 **(g).** Still images start at 50 seconds. sCSD7 also depicted in Supplementary Movie 11 and Fig. 3d-e.

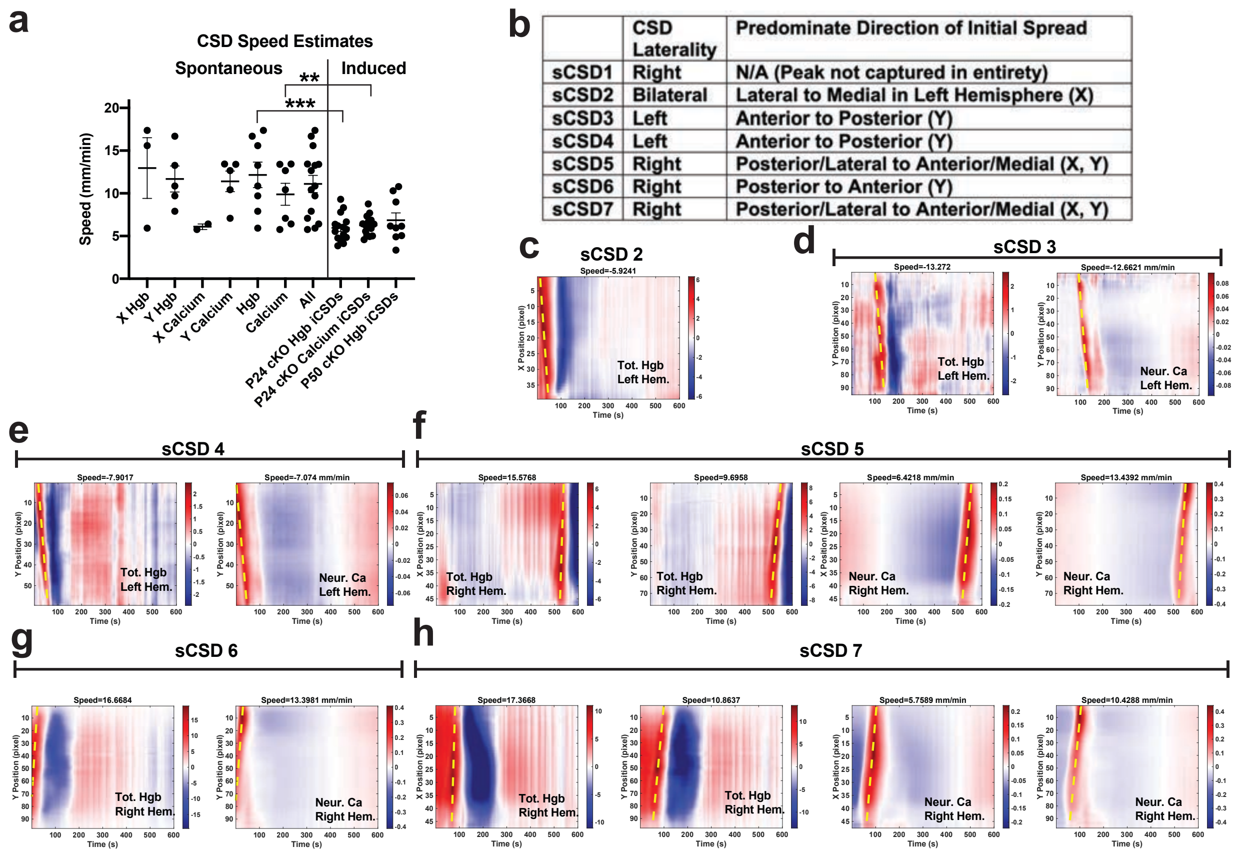


| | CSD Laterality | Predominate Direction of Initial Spread |
|---|---|---|
| sCSD1 | Right | N/A (Peak not captured in entirety) |
| sCSD2 | Bilateral | Lateral to Medial in Left Hemisphere (X) |
| sCSD3 | Left | Anterior to Posterior (Y) |
| sCSD4 | Left | Anterior to Posterior (Y) |
| sCSD5 | Right | Posterior/Lateral to Anterior/Medial (X, Y) |
| sCSD6 | Right | Posterior to Anterior (Y) |
| sCSD7 | Right | Posterior/Lateral to Anterior/Medial (X, Y) |

**Supplementary Fig 8 Estimated speed of spontaneous cortical spreading depression.**

**a-h.** Estimated CSD speeds are shown for all sCSDs and iCSDs (**a**). Data are presented as mean ± s.e.m. sCSDs were significantly faster than iCSDs at P24 in the total hemoglobin and neuronal calcium signals. **P<0.01, ***P<0.001. (Two-tailed *t* test, n=7 sCSDs total, p<0.0001 total hgb and p=0012 neuronal calcium). Since sCSDs arise from anywhere and spread any direction, we first viewed videos and qualitatively determined the hemisphere of the CSD and whether it spread in the x-axis, y-axis or both (**b**). We then created position-time plots for the axis or axes of spread in the appropriate hemisphere (**c-h**). A linear regression was then used to fit a line to the peak of the signal (yellow dotted line) The slope of these lines was used to estimate CSD speed, which is shown above each position-time plot. Each sCSD is also viewable in Supplementary Videos 5-11.

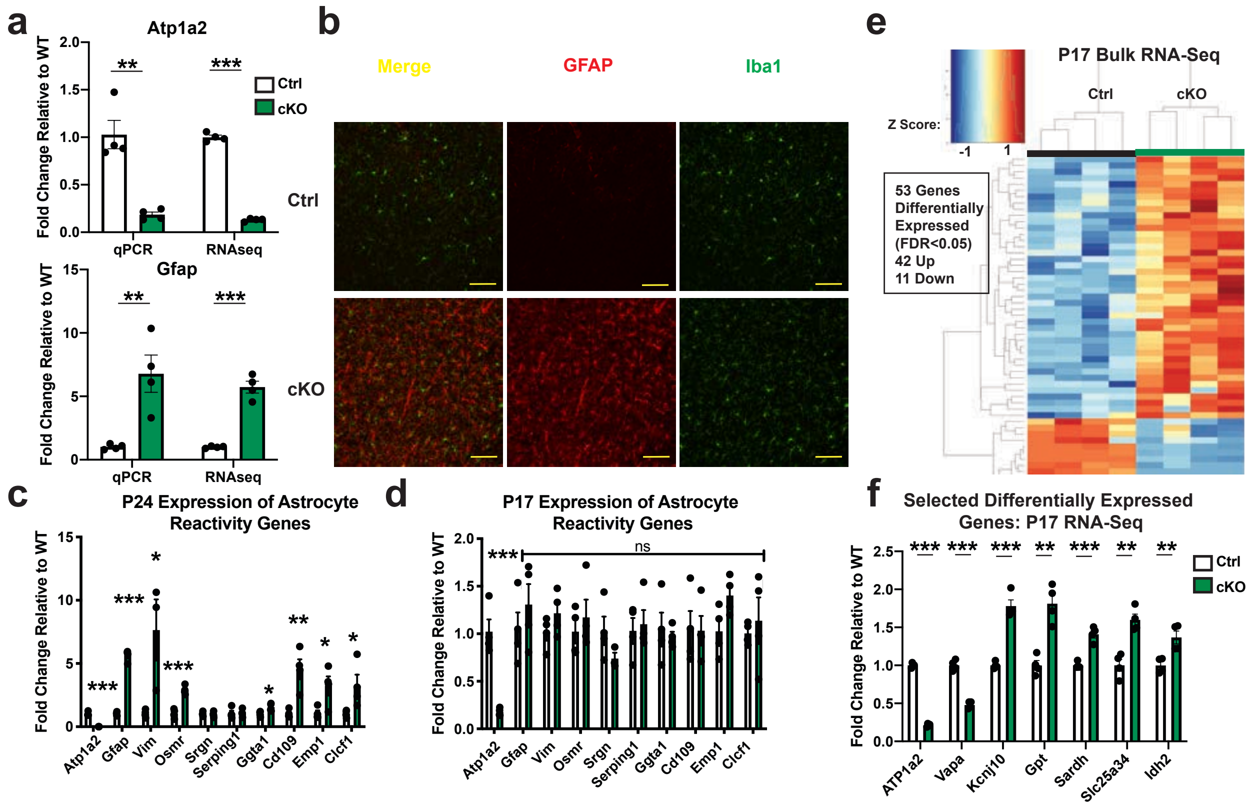


**Supplementary Fig 9 mRNA and protein expression of the cerebral cortex in conditional a2-Na/K ATPase mice suggests astrocytosis.**

**a.** The cerebral cortex from P24 conditional α2-Na/K ATPase knockout (cKO) and sex-matched littermate f/f or f/+ control (Ctrl) mice were subjected to RNA-Seq analyses (n=4). Validation of RNA-Seq relative to qRT-PCR. Fold change calculated from CPM in RNA-Seq. Fold change is normalized to Gapdh expression for qRT-PCR. Data are presented as mean ± s.e.m. **P<0.01, ***P<0.001. (Two-tailed *t* test, n=4 mice per group).

**b.** The cerebral cortex from P44-P57 cKO and Ctrl mice were subjected to immunostaining with microglial marker *Iba1* (green) and astrocyte reactivity marker *GFAP* (magenta). Representative image shown. Scale bar represents 100µm (n=3).

**c-e.** The cerebral cortex from cKO and Ctrl mice were subjected to qRT-PCR analyses at P24 **(c)** and P17 (**d**). Raw Gapdh levels did not differ significantly between groups at any timepoint (see

Supplementary Fig 14b-c). Data are presented as mean ± s.e.m. *P<0.05, **P<0.01, ***P<0.001 (Two-tailed *t* test, n=4 mice per group).

**e-f.** The cerebral cortex from P17 cKO and Ctrl mice were subjected to RNA-Seq analyses. Clustering dendrogram and heatmap of the expression levels of significantly up- and down-regulated genes. [false discovery rate (FDR)<0.05, n=4 mice per group, Z score of base 2 log-transformed CPM] (**e**). Fold change of selected significantly differentially expressed genes. Fold change calculated from CPM in RNA-Seq. Data are presented as mean ± s.e.m. **P<0.01, ***P<0.001. (Two-tailed *t* test, n=4 mice per group) (**f**).

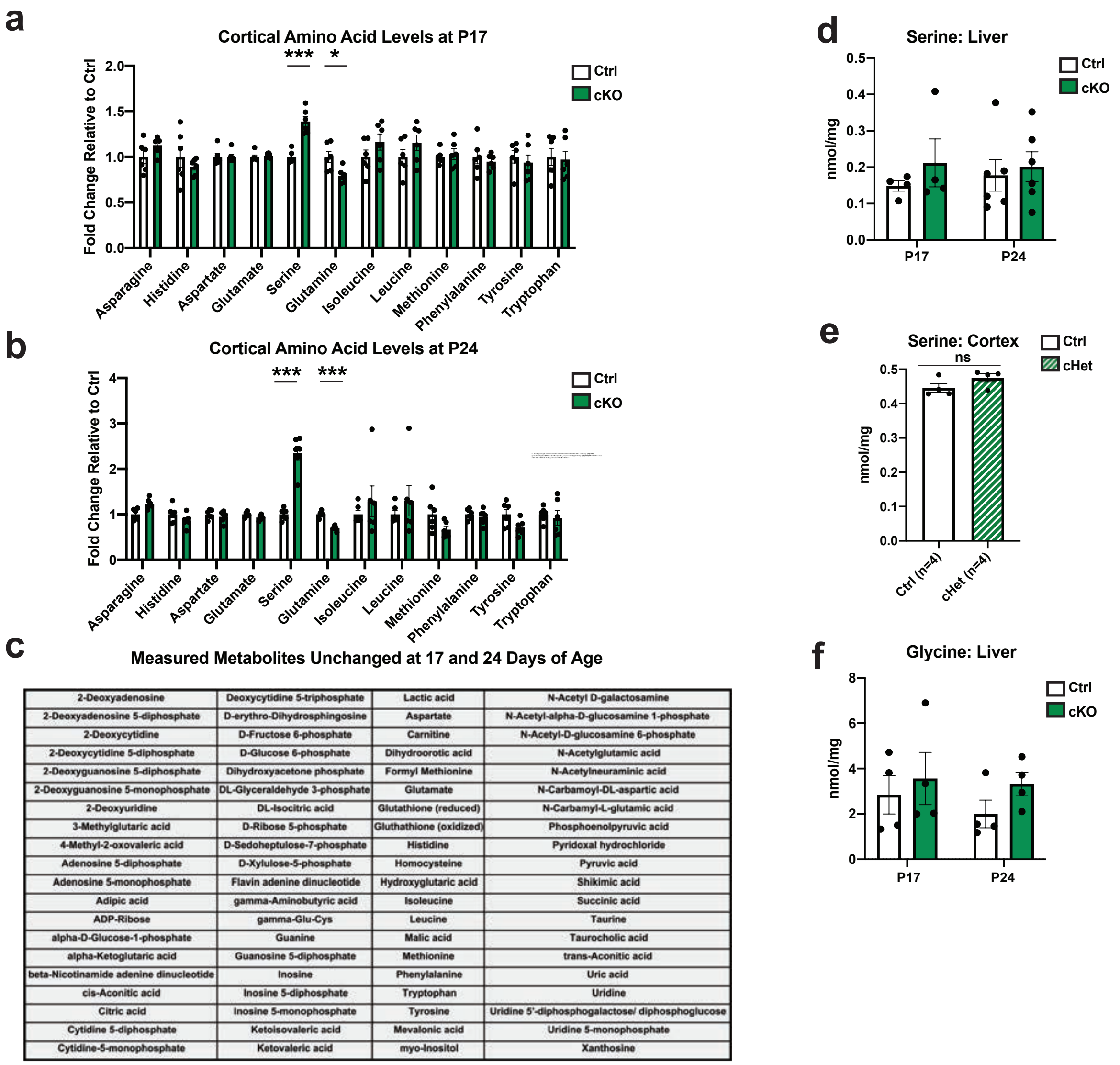


| | | | |
|---|---|---|---|
| 2-Deoxyadenosine | Deoxycytidine 5-triphosphate | Lactic acid | N-Acetyl D-galactosamine |
| 2-Deoxyadenosine 5-diphosphate | D-erythro-Dihydrosphingosine | Aspartate | N-Acetyl-alpha-D-glucosamine 1-phosphate |
| 2-Deoxycytidine | D-Fructose 6-phosphate | Carnitine | N-Acetyl-D-glucosamine 6-phosphate |
| 2-Deoxycytidine 5-diphosphate | D-Glucose 6-phosphate | Dihydroorotic acid | N-Acetylglutamic acid |
| 2-Deoxyguanosine 5-diphosphate | Dihydroxyacetone phosphate | Formyl Methionine | N-Acetylneuraminic acid |
| 2-Deoxyguanosine 5-monophosphate | DL-Glyceraldehyde 3-phosphate | Glutamate | N-Carbamoyl-DL-aspartic acid |
| 2-Deoxyuridine | DL-Isocitric acid | Glutathione (reduced) | N-Carbamyl-L-glutamic acid |
| 3-Methylglutaric acid | D-Ribose 5-phosphate | Gluthathione (oxidized) | Phosphoenolpyruvic acid |
| 4-Methyl-2-oxovaleric acid | D-Sedoheptulose-7-phosphate | Histidine | Pyridoxal hydrochloride |
| Adenosine 5-diphosphate | D-Xylulose-5-phosphate | Homocysteine | Pyruvic acid |
| Adenosine 5-monophosphate | Flavin adenine dinucleotide | Hydroxyglutaric acid | Shikimic acid |
| Adipic acid | gamma-Aminobutyric acid | Isoleucine | Succinic acid |
| ADP-Ribose | gamma-Glu-Cys | Leucine | Taurine |
| alpha-D-Glucose-1-phosphate | Guanine | Malic acid | Taurocholic acid |
| alpha-Ketoglutaric acid | Guanosine 5-diphosphate | Methionine | trans-Aconitic acid |
| beta-Nicotinamide adenine dinucleotide | Inosine | Phenylalanine | Uric acid |
| cis-Aconitic acid | Inosine 5-diphosphate | Tryptophan | Uridine |
| Citric acid | Inosine 5-monophosphate | Tyrosine | Uridine 5'-diphosphogalactose/ diphosphoglucose |
| Cytidine 5-diphosphate | Ketoisovaleric acid | Mevalonic acid | Uridine 5-monophosphate |
| Cytidine-5-monophosphate | Ketovaleric acid | myo-Inositol | Xanthosine |

**Supplementary Fig 10 Serine and glycine are altered selectively in the cortices of conditional α2-Na/K ATPase knockout mice.**

**a-c.** Mass spectrometry was performed on flash frozen cerebral cortex from P17 and P24 conditional α2-Na/K ATPase knockout (cKO) and sex-matched littermate f/f or f/+ control (Ctrl) mice. All tested amino acids at P17 **(a).** Data are presented as mean ± s.e.m. *P<0.05, ***P<0.001 (*t* test, n=6 mice per group). All tested amino acids at P24 **(b).** Data are presented as mean ± s.e.m. ***P<0.001 (Two-tailed *t* test, n=6 mice per group). Other significantly altered

metabolites are shown in Fig 5a-b. List of all metabolites that were tested but were not significant ($p>0.05$) at both timepoints **(c).**

**d.** GC-MS for serine was performed on perfused liver from P17 and P24 conditional α2-Na/K ATPase knockout (cKO) and sex-matched littermate f/f or f/+ control (Ctrl) mice (Two-tailed *t* test, $p>0.05$ at P17 and P24, n=4 mice at P17 p=0.39, 6 at P24 p=0.70).

**e.** GC-MS for serine was performed on perfused cortex from P24 conditional α2-Na/K ATPase heterozygotes (cHet) and sex-matched littermate f/f or f/+ control (Ctrl) mice (Two-tailed *t* test, $p>0.05$ at P24, n=4 mice, p=0.15).

**f.** GC-MS for glycine was performed on perfused liver from P17 and P24 conditional α2-Na/K ATPase knockout (cKO) and sex-matched littermate f/f or f/+ control (Ctrl) mice (Two-tailed *t* test, $p>0.05$ at P17 and P24, n=4 mice per group, p=0.63 P17, p=0.15 P24).

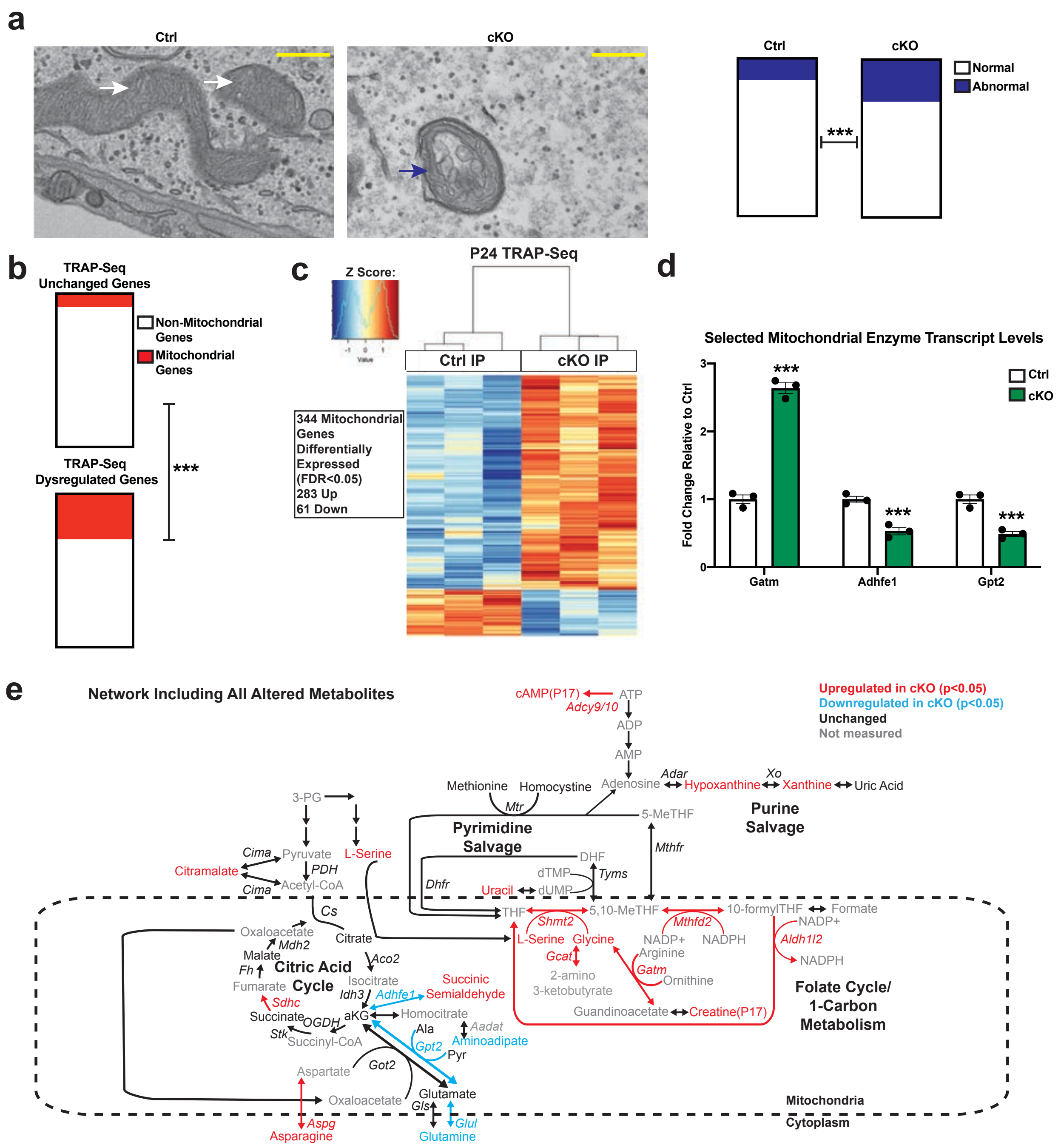


**Supplementary Fig 11 Mitochondrial morphology and gene expression is altered in the cortices of conditional astrocytic α2-Na/K ATPase knockout mice.**

**a.** Ultrastructural appearance of astrocytic mitochondria in conditional α2-Na/K ATPase knockout mice is significantly abnormal. Electron microscopy example images (left) and quantification (right) of astrocytic mitochondria from layer 2/3 of motor cortex in P24

conditional α2-Na/K ATPase knockout mice (cKO) and littermate sex-matched f/f or f/+ controls (Ctrl). Abnormal vs. normal astrocytic mitochondria were judged by an experienced, blinded observer (white arrows refer to normal mitochondria examples, blue arrows refer to abnormal mitochondria example). Scale bar is 500nm. Abnormal and normal mitochondria were counted for each mouse. ***P<0.001 (Chi-squared test, n=719 total mitochondria in Ctrl group and 2286 total mitochondria in cKO group from 5 mice per group, p<0.0001)

**b-d.** TRAP-Seq data shows enrichment for mitochondrial genes, which are predominately upregulated. Genes that were differentially expressed at P24 in the cortical astrocytes of conditional α2-Na/K ATPase knockout mice using TRAP-Seq were intersected with mitochondrial genes, and showed significant enrichment of mitochondrial genes among differentially expressed genes in the TRAP-Seq (**b**). ***P<0.001 (Chi-squared test, n=3435 genes dysregulated in TRAP-Seq and n=52999 genes not dysregulated from 3 mice per group, p<0.0001). Clustering dendrogram and heatmap of the expression levels of significantly up- and down-regulated genes among mitochondrial genes dysregulated in the TRAP-Seq dataset (**c**) (false discovery rate (FDR)<0.05, n=3 mice per group, Z score of base 2 log-transformed CPM). Fold change of selected mitochondrial enzymes in the TRAP-Seq for conditional α2-Na/K ATPase knockout mice (cKO) relative to sex-matched littermate f/f or f/+ control (Ctrl) mice (**d**). Data are presented as mean ± s.e.m. ***P<0.001 (False discovery rate (FDR), n=3 mice per group).

**e.** Intersection of the TRAP-Seq data for transcript levels of metabolic enzymes with the metabolomics for metabolite levels using all significantly altered metabolites in data set at P17 and P24 (if only altered at P17, metabolite is labeled P17). Red indicates significant upregulation

of the transcript or metabolite ($p<0.05$), blue indicates significant downregulation, black indicates no significant change and gray indicates an unmeasured metabolite.

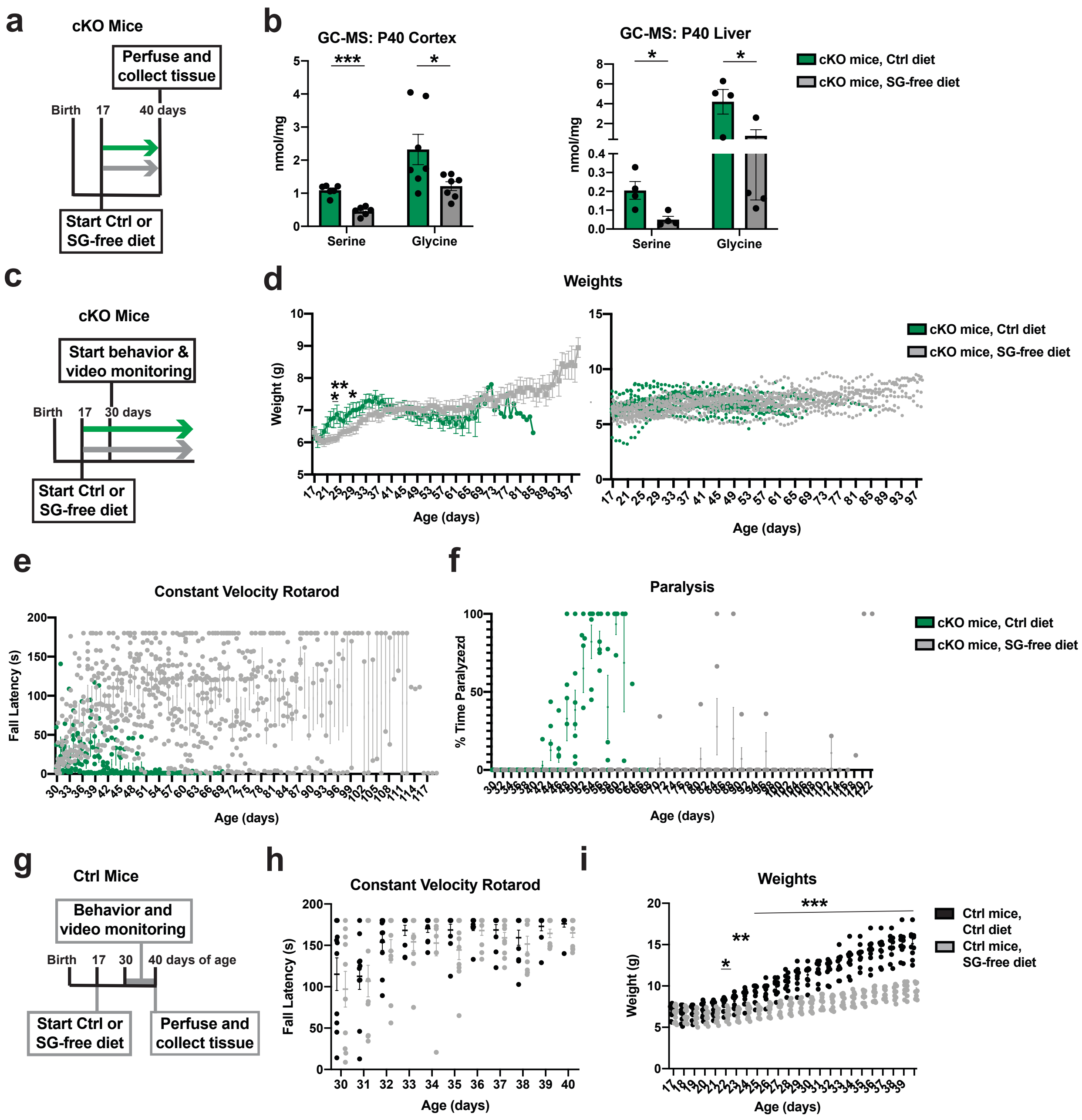


**Supplementary Fig 12 Administering a serine- and glycine-free diet in control and conditional α2-Na/K ATPase knockout mice.**

**a-b.** Sex-matched littermate conditional α2-Na/K ATPase knockout (cKO) mice were weaned onto control (Ctrl) or serine- and glycine-free (SG-free) diet at P17 and weighed daily thereafter. Sex-matched littermate P40 conditional α2-Na/K ATPase knockout (cKO) mice on Ctrl and SG-

free food were perfused and the cerebral cortex or liver was subjected to GC-MS for serine and glycine levels **(b).** Data are presented as mean ± s.e.m. *P<0.05, ***P<0.001 (Two-tailed *t* test, n=6 mice per group for serine cortex, 7 mice per group for glycine cortex, 4 mice per group for serine and glycine liver).

**c-f.** Sex-matched littermate conditional α2-Na/K ATPase knockout (cKO) mice were weaned onto ctrl or serine- and glycine-free (SG-free) diet at P17 and weighed daily thereafter. Rotarod and paralysis monitoring started at P30, with rotarod testing occurring daily and paralysis monitoring occurring every other day. Complete data shown for every mouse until its death or humane end-point (n=17 mice per group starting at P17, Ctrl: 16 at P23, 15 at P24, 13 at P27, 12 at P43, 11 at P51, 10 at P54, 9 at P58, 7 at P62, 5 at P64, 3 at P66, 2 at P69, 1 at P70, 0 at P86, SG-Free: 16 at P23, 15 at P31, 14 at P32, 13 at P56, 12 at P61, 11 at P67, 10 at P75, 9 at P77, 8 at P78, 7 at P86, 6 at P92, 5 at P94, 4 at P99, 3 at P103, 2 at P113, 1 at P123). Weight of sex-matched littermate conditional α2-Na/K ATPase knockout (cKO) mice on ctrl or serine- and glycine-free (SG-free) diet (**d**). Data are presented as mean ± s.e.m on the left, and all data points are shown on the right. *P<0.05, **P<0.01 (Two-way ANOVA followed by Fisher's LSD *post hoc* test). Complete data for all mice up to their endpoint - mean latency to fall on constant velocity rotarod task for sex-matched littermate conditional α2-Na/K ATPase knockout (cKO) mice on ctrl or SG-free diet (**e**) Data are presented as mean ± s.e.m. Complete data for all mice up to their endpoint - percentage of time paralyzed for sex-matched littermate conditional α2-Na/K ATPase knockout mice (cKO) on Ctrl or SG-free food (**f**) Data are presented as mean ± s.e.m.

**g-i.** Sex-matched littermate f/f or f/+ control (Ctrl) mice were weaned at P17 onto control (Ctrl) or serine- and glycine-free (SG-free) diet. Rotarod and paralysis monitoring started at P30, with

rotarod testing occurring daily and paralysis monitoring occurring every other day. Ctrl mice exhibited no paralysis (data not shown). Mice were perfused at P40 and tissues collected. Mean latency to fall on constant velocity rotarod task for sex-matched littermate f/f or f/+ control (Ctrl) mice on Ctrl or SG-free diet **(h).** Data are presented as mean ± s.e.m. No significant difference. (Two-way ANOVA followed by Fisher's LSD *post hoc* test, n=10 mice per group). Weight of sex-matched littermate f/f or f/+ control (Ctrl) mice on ctrl or SG-free food **(i).** Data are presented as mean ± s.e.m. *P<0.05, **P<0.01, ***P<0.001 (Two-way ANOVA followed by Fisher's LSD *post hoc* test, n=10 mice per group).

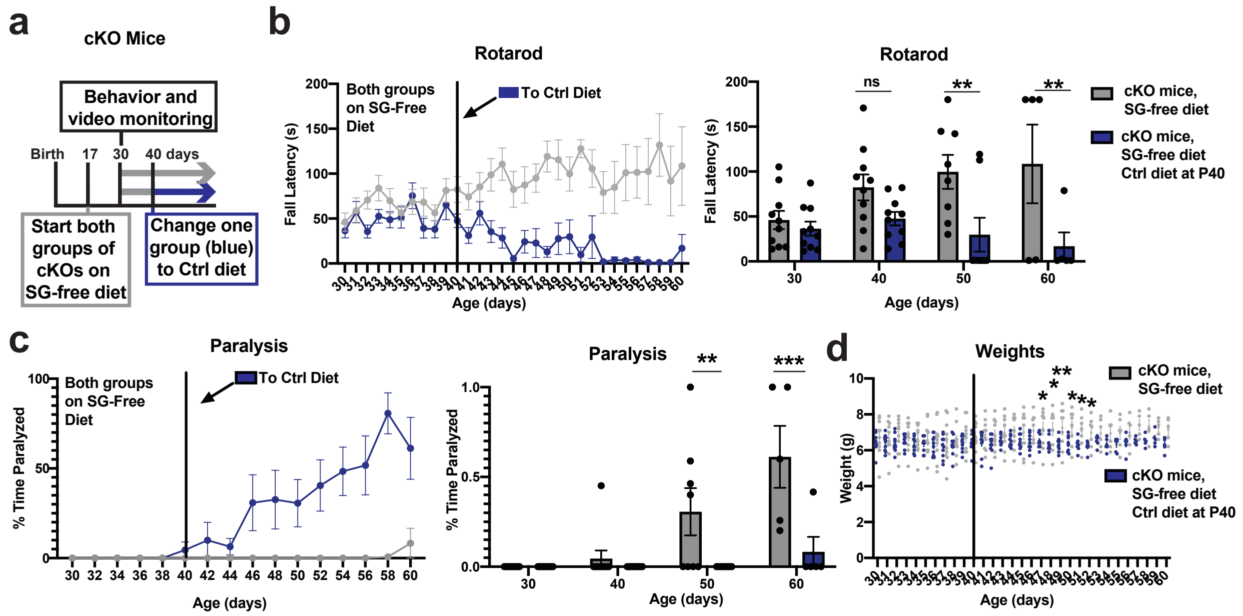


**Supplementary Fig 13 Effects of the Serine- and Glycine-free Diet on Motor Paralysis and Ataxia in Conditional α2-Na/K ATPase Knockout Mice is Reversible.**

**a-c.** Sex-matched littermate conditional α2-Na/K ATPase knockout (cKO) mice were weaned onto serine- and glycine-free (SG-free) diet at P17 and weighed daily thereafter. Daily rotarod analyses and every other day monitoring for motor paralysis of mice was begun at P30. One group was changed to control (Ctrl) diet at P40, the other group was left on serine- and glycine-free (SG-free) diet. Left: Mean latency to fall on constant velocity rotarod task for sex-matched littermate conditional α2-Na/K ATPase knockout (cKO) mice on SG-free diet the whole time or Ctrl diet from P40 **(b).** Data are presented as mean ± s.e.m. (n=10 mice per group starting at P17, 9 at P43, 8 at P50, 7 at P51, 6 at P53, 5 at P58). Right: A subset of data from left side shown for clarity. Data are presented as mean ± s.e.m. **P<0.01 (Two-way ANOVA followed by Fisher's LSD *post hoc* test, n=10 mice per group starting at P17, 8 mice per group at P50 and 5 at P60). Left: Percentage of time paralyzed for sex-matched littermate conditional α2-Na/K ATPase knockout (cKO) mice on SG-free diet the whole time or Ctrl diet from P40 **(c).** Data are

presented as mean ± s.e.m. **P<0.01, ***P<0.001 (n=10 mice per group starting at P17, 9 at P43, 8 at P50, 7 at P51, 6 at P53, 5 at P58). Right: A subset of data from left side shown for clarity. Data are presented as mean ± s.e.m. **P<0.01, ***P<0.001 (Two-way ANOVA followed by Fisher's LSD *post hoc* test, n=10 mice per group starting at P17, 9 at P43, 8 at P50, 7 at P51, 6 at P53, 5 at P58).

**d.** Weight of sex-matched littermate conditional α2-Na/K ATPase knockout (cKO) mice on SG-free diet the whole time or Ctrl diet from P40**.** Data are presented as mean ± s.e.m. *P<0.05, **P<0.01 (Two-way ANOVA followed by Fisher's LSD *post hoc* test, n=10 mice per group starting at P17, 9 at P43, 8 at P50, 7 at P51, 6 at P53, 5 at P58).

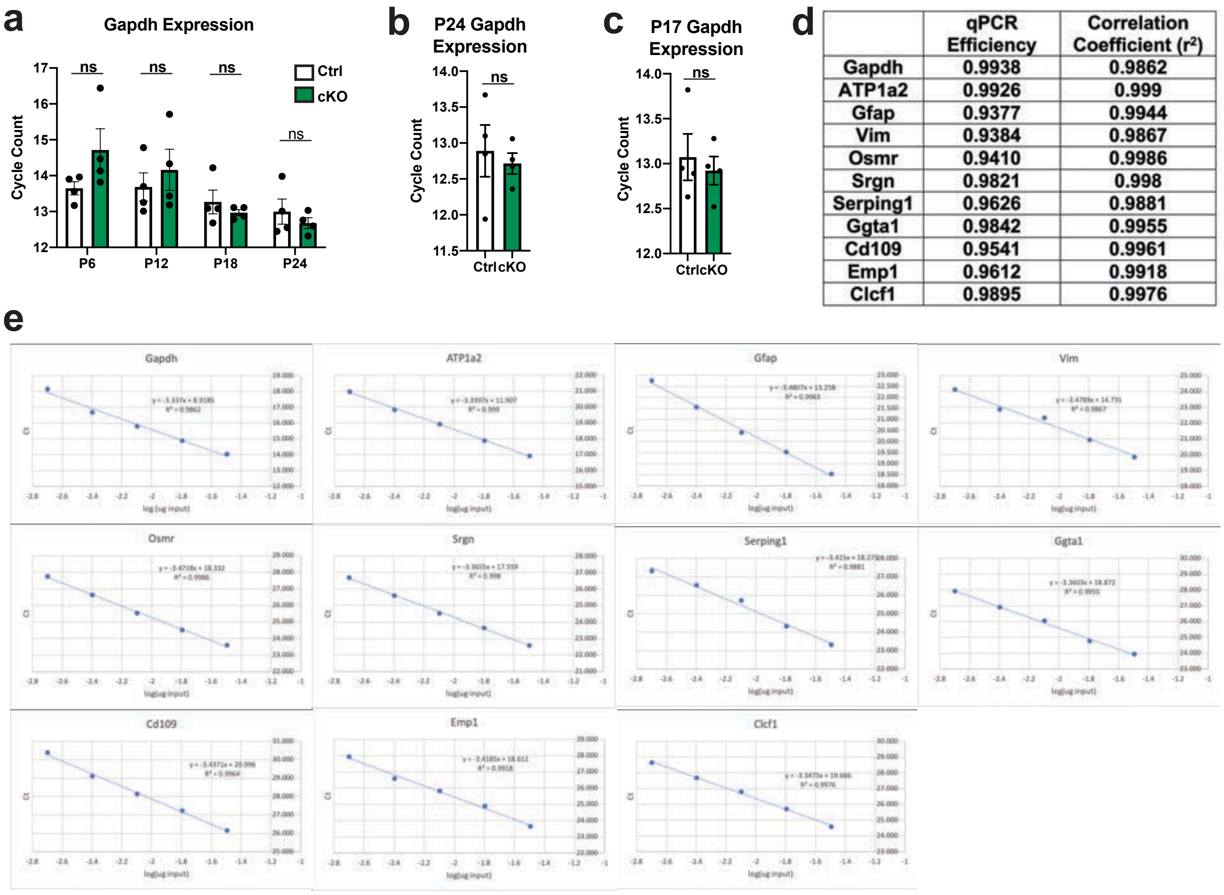


| | qPCR Efficiency | Correlation Coefficient ($r^2$) |
|---|---|---|
| Gapdh | 0.9938 | 0.9862 |
| ATP1a2 | 0.9926 | 0.999 |
| Gfap | 0.9377 | 0.9944 |
| Vim | 0.9384 | 0.9867 |
| Osmr | 0.9410 | 0.9986 |
| Srgn | 0.9821 | 0.998 |
| Serping1 | 0.9626 | 0.9881 |
| Ggta1 | 0.9842 | 0.9955 |
| Cd109 | 0.9541 | 0.9961 |
| Emp1 | 0.9612 | 0.9918 |
| Clcf1 | 0.9895 | 0.9976 |

**Supplementary Fig 14 Standard Curves for qRT-PCR Primer Pairs.**

**a.** Raw Ct value for Gapdh in the data shown in Fig 1b. Data are presented as mean ± s.e.m. ns: P>0.05 (Two-way ANOVA followed by Fisher's LSD *post hoc* test, n=4 mice per group).

**b.** Raw Ct value for Gapdh in the data shown in Supplementary Fig 9c. Data are presented as mean ± s.e.m. ns: P>0.05 (Two-way ANOVA followed by Fisher's LSD *post hoc* test, n=4 mice per group, p=0.668).

**c.** Raw Ct value for Gapdh in the data shown in Supplementary Fig 9d. Data are presented as mean ± s.e.m. ns: P>0.05 (Two-way ANOVA followed by Fisher's LSD *post hoc* test, n=4 mice per group, p=0.637).

**d.** Table of calculated PCR efficiency and correlation coefficient ($r^2$) for each primer pair used in qRT-PCR experiments.

**e.** Experimental standard curve and fit line for each primer pair.

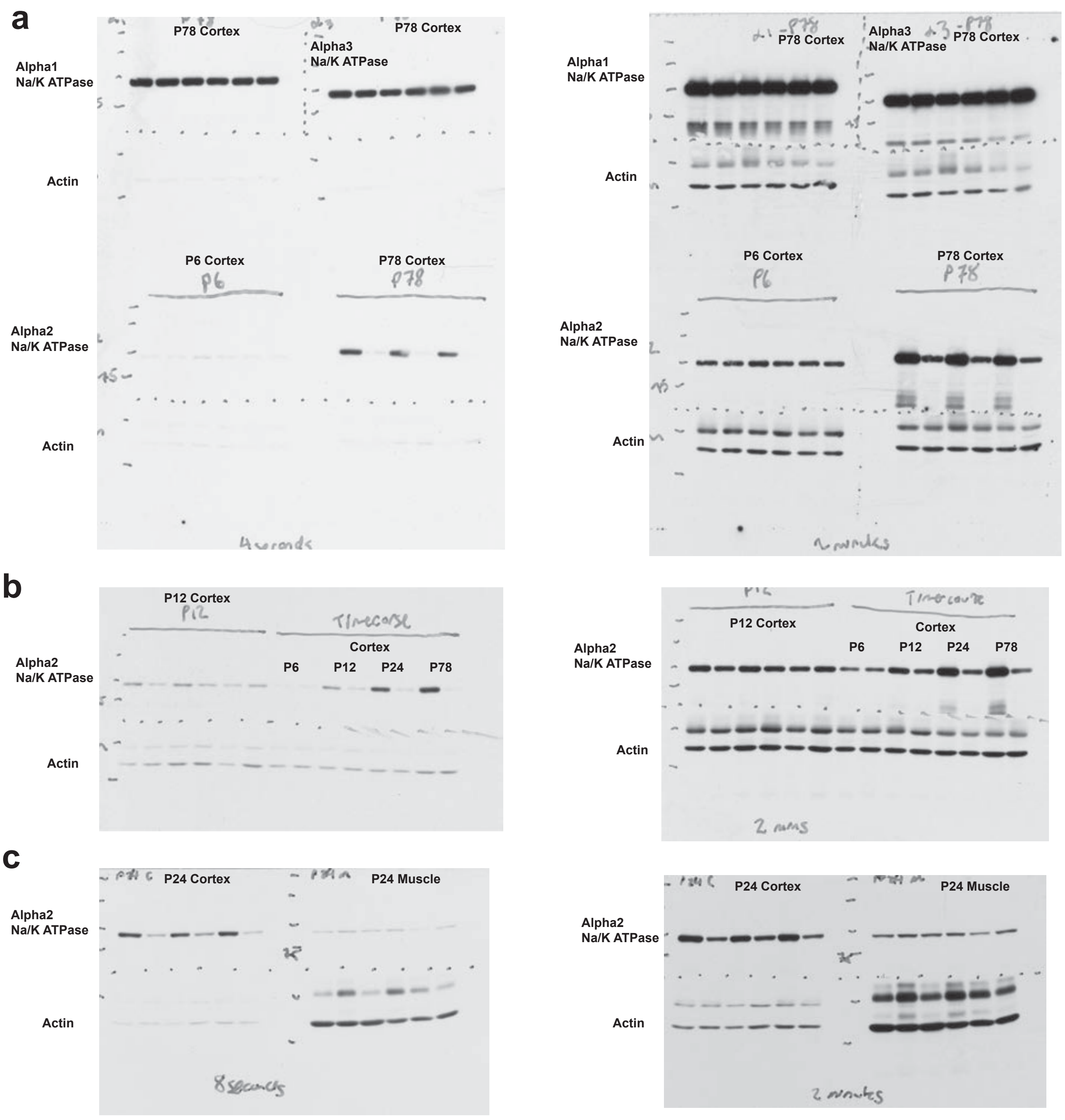


**Supplementary Fig 15 Full scanned blots for all immunoblot experiments.**

**a-c.** For each blot, the ladder lines are, in order from top to bottom, 250 kDa, 150, 100, 75 (labeled), 50, 37. The dotted lines indicate where blots are cut for different antibodies. Antibodies and tissue type and age are listed on the blots. For each blot, samples are, from left to right, Ctrl, cKO, Ctrl, cKO, etc. 10ug of protein was loaded. Exposure times were 4 seconds with

the blots on the left side and 2 minutes for the blots on the right side for (**a-b)**, and 8 seconds with the blots on the left side and 2 minutes for the blots on the right side for (**c)**. See Supplementary Fig 2 for cropped and labeled blots with quantification.
