# Supplementary material for: Astrocyte deletion of α2-Na/K ATPase triggers episodic motor paralysis in mice via a metabolic pathway
Source: Nat Commun. 2020 Dec 2;11:6164. doi: 10.1038/s41467-020-19915-2 (PMC7710756; doi:10.1038/s41467-020-19915-2)
Supplement: Supplementary file 2 — Description of Additional Supplementary Files [file 41467_2020_19915_MOESM2_ESM.pdf]

Description of Additional Supplementary Files

Supplementary Movie 1

Representative example of transient paralysis. P50 conditional male α2-Na/K ATPase knockout mouse during the middle of an episode of transient paralysis.

Supplementary Movie 2

Transient paralysis recovery. P50 female conditional α2-Na/K ATPase knockout mouse at the very end of an episode of transient paralysis. The mouse previously had a phenotype similar to the mouse in Supplementary Movie 1. The mouse now is recovering from the episode, regaining more normal motor function and transitioning to ambulation.

Supplementary Movie 3

Normal Ambulation - Male. P50 conditional male α2-Na/K ATPase knockout mouse between episodes of transient paralysis, exhibiting normal ambulation.

Supplementary Movie 4

Normal Ambulation - Female. P50 conditional female α2-Na/K ATPase knockout mouse between episodes of transient paralysis, exhibiting normal ambulation.

Supplementary Movie 5

Spontaneous cortical spreading depression 1. Movie is 10x real time speed. Right: EEG from right hemisphere. Left: Total Hemoglobin (Tot. Hgb), scale bar units are Δ(uM) hemoglobin.

Supplementary Movie 6

Spontaneous cortical spreading depression 2. Movie is 10x real time speed. Right: EEG from right hemisphere. Left: Total Hemoglobin (Tot. Hgb), scale bar units are Δ(uM) hemoglobin.

Supplementary Movie 7

Spontaneous cortical spreading depression 3. Movie is 10x real time speed. Right: Neuronal calcium signal (from Thy1- GCaMP6f), scale bar units are ΔF/F. Left: Total Hemoglobin (Tot. Hgb), scale bar units are Δ(uM) hemoglobin.

Supplementary Movie 8

Spontaneous cortical spreading depression 4. Movie is 10x real time speed. Right: Neuronal calcium signal (from Thy1- GCaMP6f), scale bar units are ΔF/F. Left: Total Hemoglobin (Tot. Hgb), scale bar units are Δ(uM) hemoglobin.

Supplementary Movie 9

Spontaneous cortical spreading depression 5. Movie is 10x real time speed. Right: EEG from right hemisphere. Middle: Neuronal calcium signal (from Thy1-GCaMP6f), scale bar units are ΔF/F. Left: Total Hemoglobin (Tot. Hgb), scale bar units are Δ(uM) hemoglobin.

Supplementary Movie 10

Spontaneous cortical spreading depression 6. Movie is 10x real time speed. Right: EEG from right hemisphere. Middle: Neuronal calcium signal (from Thy1-GCaMP6f), scale bar units are ΔF/F. Left: Total Hemoglobin (Tot. Hgb), scale bar units are Δ(uM) hemoglobin.

Supplementary Movie 11

Spontaneous cortical spreading depression 7. Movie is 10x real time speed. Right: EEG from right hemisphere. Middle: Neuronal calcium signal (from Thy1-GCaMP6f), scale bar units are ΔF/F. Left: Total Hemoglobin (Tot. Hgb), scale bar units are Δ(uM) hemoglobin.

Supplementary Data 1

Differential gene expression for RNA-sequencing experiments and metabolomics source data. Differential RNA gene expression in P24 Bulk RNA-Seq (Sheet 1), P17 Bulk RNA-Seq (Sheet 2) and P24 TRAP-Seq (Sheet 3). For each experiment, for the significantly (FDR<0.05) differentially expressed genes, the gene ID, log2 fold change in expression, log2 mean counts per million, p value, false discovery rate (FDR, adjusted p value) and the gene name is shown. For P23 TRAP-Seq genes, significantly altered mitochondrial genes are also highlighted. Genes are sorted by up- and down-regulated genes in the cKO samples. Metabolomics source data is provided (Sheet 4).

Supplementary Data 2

Statistical Details.
