# Supplementary material for: Astrocyte deletion of α2-Na/K ATPase triggers episodic motor paralysis in mice via a metabolic pathway
Source: Nat Commun. 2020 Dec 2;11:6164. doi: 10.1038/s41467-020-19915-2 (PMC7710756; doi:10.1038/s41467-020-19915-2)
Supplement: Supplementary file 16 — Reporting Summary [file 41467_2020_19915_MOESM16_ESM.pdf]

nature research

Corresponding author(s): Azad Bonni

Last updated by author(s): Oct 27, 2020

Reporting Summary

Nature Research wishes to improve the reproducibility of the work that we publish. This form provides structure for consistency and transparency in reporting. For further information on Nature Research policies, see Authors & Referees and the Editorial Policy Checklist .

Statistics

For all statistical analyses, confirm that the following items are present in the figure legend, table legend, main text, or Methods section.

n/a	Confirmed	
☐	☒	The exact sample size (*n*) for each experimental group/condition, given as a discrete number and unit of measurement
☐	☒	A statement on whether measurements were taken from distinct samples or whether the same sample was measured repeatedly
☐	☒	The statistical test(s) used AND whether they are one- or two-sided<br>*Only common tests should be described solely by name; describe more complex techniques in the Methods section.*
☐	☒	A description of all covariates tested
☐	☒	A description of any assumptions or corrections, such as tests of normality and adjustment for multiple comparisons
☐	☒	A full description of the statistical parameters including central tendency (e.g. means) or other basic estimates (e.g. regression coefficient) AND variation (e.g. standard deviation) or associated estimates of uncertainty (e.g. confidence intervals)
☐	☒	For null hypothesis testing, the test statistic (e.g. F, t, r) with confidence intervals, effect sizes, degrees of freedom and P value noted<br>*Give P values as exact values whenever suitable.*
☒	☐	For Bayesian analysis, information on the choice of priors and Markov chain Monte Carlo settings
☒	☐	For hierarchical and complex designs, identification of the appropriate level for tests and full reporting of outcomes
☒	☐	Estimates of effect sizes (e.g. Cohen's *d*, Pearson's *r*), indicating how they were calculated

Our web collection on statistics for biologists contains articles on many of the points above.

Software and code

Policy information about availability of computer code

Data collection	Custom matlab code was used to acquire data for Intrinsic Optical Signal (iOS) Imaging, Wide-Field Calcium Imaging and EEG recording, which has previously been described in published literature. We have provided this code on github (https://github.com/brierl/Nature_Comm_CSD).
Data analysis	Custom matlab code was used to analyze data for Intrinsic Optical Signal (iOS) Imaging, Wide-Field Calcium Imaging and EEG recording, which has previously been described in published literature. We have provided this code on github (https://github.com/brierl/Nature_Comm_CSD).<br>RNA-Seq Galaxy Software: Star (v2.6.0b), Samtools (v1.8), Bedtools (v2.27.0)<br>RNA-Seq R Software: EdgeR (v3.30.3)<br>Statistical analyses were done using GraphPad Prism software V7 or V8.<br>Image analysis performed in FIJI v2.0.0-rc-69/1.52n.<br>Data organization performed in Microsoft Excel 365.

For manuscripts utilizing custom algorithms or software that are central to the research but not yet described in published literature, software must be made available to editors/reviewers. We strongly encourage code deposition in a community repository (e.g. GitHub). See the Nature Research guidelines for submitting code & software for further information.

Data

Policy information about availability of data

All manuscripts must include a data availability statement. This statement should provide the following information, where applicable:

- Accession codes, unique identifiers, or web links for publicly available datasets
- A list of figures that have associated raw data
- A description of any restrictions on data availability

All sequencing data is deposited into GEO with accession code GSE145012 [https://www.ncbi.nlm.nih.gov/geo/query/acc.cgi?acc=GSE145102]. Differentially

expressed genes and metabolomics data are provided in Supplementary Data 1. Statistical details for all statistical tests including exact p values included in Supplementary Data 2. All data supporting our findings can be found within the article and its supplementary information. Source Data file is provided with this article.

Field-specific reporting

Please select the one below that is the best fit for your research. If you are not sure, read the appropriate sections before making your selection.

- [x] Life sciences
- [ ] Behavioural & social sciences
- [ ] Ecological, evolutionary & environmental sciences

For a reference copy of the document with all sections, see nature.com/documents/nr-reporting-summary-flat.pdf

Life sciences study design

All studies must disclose on these points even when the disclosure is negative.

Sample size	No statistical methods were used to pre-determine sample sizes, but our sample sizes are similar to those generally employed in the field. Standards of the field (e.g. https://www.encodeproject.org/about/experiment-guidelines/ for sequencing experiments) were used to determine the number of biological replicates per condition.
Data exclusions	No data were excluded from the analyses
Replication	The reproducibility of biological replicates were assessed by standard methods. The clustering of individual biological replicates are reported in manuscript. For all experiments, 2-3+ biological replicates were used per condition. All replicates were successful.
Randomization	Mice were randomly allocated into groups e.g. different diets.
Blinding	For behavioral experiments or experiments where subjective measurements are made, experiments were conducted with the experimenter blinded to the group of the mouse. Otherwise, experimenters were not blinded to experimental conditions since no subjective measurements were made.

Reporting for specific materials, systems and methods

We require information from authors about some types of materials, experimental systems and methods used in many studies. Here, indicate whether each material, system or method listed is relevant to your study. If you are not sure if a list item applies to your research, read the appropriate section before selecting a response.

Materials & experimental systems

n/a	Involved in the study	
[ ]	[x]	Antibodies
[x]	[ ]	Eukaryotic cell lines
[x]	[ ]	Palaeontology
[ ]	[x]	Animals and other organisms
[x]	[ ]	Human research participants
[x]	[ ]	Clinical data

Methods

n/a	Involved in the study	
[x]	[ ]	ChIP-seq
[x]	[ ]	Flow cytometry
[x]	[ ]	MRI-based neuroimaging

Antibodies

Antibodies used	Antibodies to α2-Na/K ATPase (Immunoblot, 1:20,000, Millipore, AB9094), α1-Na/K ATPase (Immunoblot, 1:5,000, Thermo Fisher, MA3-928), α3-Na/K ATPase (Immunoblot, 1:5,000, Thermo Fisher, MA3-915), Actin (Immunoblot, 1:500, Santa Cruz, SC8432), GFP (Immunofluorescence, 1:500, Abcam, AB13970), NeuN (Immunofluorescence, 1:500, Abcam, AB177487), GFAP (Immunofluorescence, 1:500, Millipore, MAB3402), S100β (Immunofluorescence, 1:500, Abcam, AB41548), Iba1 (Immunofluorescence, 1:500, Wako, 019-19741), cleaved caspase-3 (Immunofluorescence, 1:500, Cell Signaling D175) and Alexa Fluor conjugated secondary antibodies (Immunofluorescence, 1:500, Invitrogen, A11039, A11004, A11001, A10042) were purchased.
Validation	All are commercial antibodies used extensively in other studies, with validation information and relevant citations for the described applications available on the manufacturers' websites.

Animals and other organisms

Policy information about studies involving animals; ARRIVE guidelines recommended for reporting animal research

Laboratory animals	Animals were Mus Musculus of the C57 Black 6 strain. Both male and female animals of various ages (P6-P110) were used, as specified in the manuscript. Mice were purchased or maintained under pathogen-free conditions. Mice were kept on a 12 on/12

	off light cycle with room temperature 20-26C and humidity 30-70%.
Wild animals	The study did not involve wild animals.
Field-collected samples	The study did not involve samples caught in the field.
Ethics oversight	All animal experiments were done according to protocols approved by the Animal Studies Committee of Washington University School of Medicine and in accordance with the National Institutes of Health guidelines.

Note that full information on the approval of the study protocol must also be provided in the manuscript.
